# Combination of T-Cell Bispecific Antibodies With PD-L1 Checkpoint Inhibition Elicits Superior Anti-Tumor Activity

**DOI:** 10.3389/fonc.2020.575737

**Published:** 2020-11-30

**Authors:** Johannes Sam, Sara Colombetti, Tanja Fauti, Andreas Roller, Marlene Biehl, Linda Fahrni, Valeria Nicolini, Mario Perro, Tapan Nayak, Esther Bommer, Anne Schoenle, Maria Karagianni, Marine Le Clech, Nathalie Steinhoff, Christian Klein, Pablo Umaña, Marina Bacac

**Affiliations:** ^1^ Roche Pharma Research & Early Development, Roche Innovation Center Zurich, Zurich, Switzerland; ^2^ Roche Pharma Research & Early Development, Roche Innovation Center Basel, Basel, Switzerland

**Keywords:** solid tumors, immunotherapy, T-cell bispecific antibody, carcinoembryonic antigen T-cell bispecific antibody, programmed death–ligand 1, combination, humanized mice

## Abstract

T-cell Bispecific Antibodies (TCBs) elicit anti-tumor responses by cross-linking T-cells to tumor cells and mediate polyclonal T-cell expansion that is independent of T-cell receptor specificity. TCBs thus offer great promise for patients who lack antigen-specific T-cells or have non-inflamed tumors, which are parameters known to limit the response of checkpoint inhibitors. The current study deepens the understanding of TCB mode of action and elaborates on one of the adaptive resistance mechanisms following its treatment *in vivo* in humanized mice and syngeneic pre-clinical tumor models. Single-agent TCB treatment reduced tumor growth compared with controls and led to a 2–10-fold increase in tumor-infiltrating T-cells, regardless of the baseline tumor immune cell infiltration. TCB treatment strongly induced the secretion of CXCL10 and increased the frequency of intra-tumor CXCR3+ T-cells pointing to the potential role of the CXCL10-CXCR3 pathway as one of the mechanisms for T-cell recruitment to tumors upon TCB treatment. Tumor-infiltrating T-cells displayed a highly activated and proliferating phenotype, resulting in the generation of a highly inflamed tumor microenvironment. A molecular signature of TCB treatment was determined (CD8, PD-1, MIP-a, CXCL10, CXCL13) to identify parameters that most robustly characterize TCB activity. Parallel to T-cell activation, TCB treatment also led to a clear upregulation of PD-1 on T-cells and PD-L1 on tumor cells and T-cells. Combining TCB treatment with anti-PD-L1 blocking antibody improved anti-tumor efficacy compared to either agent given as monotherapy, increasing the frequency of intra-tumoral T-cells. Together, the data of the current study expand our knowledge of the molecular and cellular features associated with TCB activity and provide evidence that the PD-1/PD-L1 axis is one of the adaptive resistance mechanisms associated with TCB activity. This mechanism can be managed by the combination of TCB with anti-PD-L1 blocking antibody translating into more efficacious anti-tumor activity and prolonged control of the tumor outgrowth. The elucidation of additional resistance mechanisms beyond the PD-1/PD-L1 axis will constitute an important milestone for our understanding of factors determining tumor escape and deepening of TCB anti-tumor responses in both solid tumors and hematological disorders.

## Introduction

Targeting T-cells with antibodies that directly enhance T-cell activity, including the checkpoint inhibitory molecules (CPIs) programmed death receptor 1 (PD-1), PD-ligand 1 (PD-L1), and cytotoxic lymphocyte activated antigen 4 (CTLA4) has become an established approach in clinical practice (1–3). Antibodies to checkpoint molecules have gained broad approval in various tumor indications for the treatment of advanced cancer types such as metastatic melanoma, advanced non-small cell lung cancer, or renal cell carcinoma (4). However, despite these advances, obstacles still exist including the inability to predict treatment efficacy and patient response, the need for additional biomarkers, the development of primary and secondary resistance to cancer immunotherapies, the lack of clinical study designs that are optimized to determine efficacy and toxicity (and their relationship), and high treatment costs (5).

T-cell Bispecific Antibodies (TCBs) elicit anti-tumor responses by cross-linking of T-cells to target tumor cells (6, 7). TCB-mediated polyclonal T-cell activation is independent of the T-cell receptor specificity and does not require (at least initially) costimulatory signals. Thus, factors normally affecting the efficiency of CPIs to mount an endogenous anti-tumor immune response, including MHC downregulation, antigen presentation, the frequency of antigen-specific T-cells, T-cell receptor affinity, and T-cell avidity, are less relevant for TCB activity. Hence, TCBs are a highly attractive approach to activate T-cells regardless of their antigen specificity. Due to the increase of intra-tumor T-cell infiltration upon treatment (8–10), TCBs offer great promise in patients that lack the baseline antigen-specific T-cells (or any type of T-cells, the so called immune desert tumors), which is thought to render responses to checkpoint inhibition less likely (5).

Although more than forty different bispecific antibodies have been described to date (6, 11–14), the promising results obtained in preclinical studies do not translate directly into the clinical setting. Only two TCBs were approved for use in the clinic so far: catumaxomab and blinatumomab. Catumaxomab targets epithelial cell adhesion molecule (EpCAM) and was initially approved by the European Medicines Agency (EMA) in 2009 for the treatment of malignant ovarian ascites (15), Catumaxomab has not been marketed in the EU since 2014 and market authorization was withdrawn in 2017. Blinatumomab targets CD19 and was approved by the FDA and EMA in 2014 and 2015, respectively, for the treatment of Philadelphia chromosome negative B cell acute lymphoblastic leukemia (16). Promising clinical activity has been reported with other TCBs, particularly in hematological malignancies (6, 7, 17).

Clinical development of TCBs in solid tumors has been challenging and may be hampered by multiple constraints. These include the lack of tumor-specific antigens that are not expressed in primary epithelium (18), the local suppressive tumor microenvironment [characterized by expression of IL10, TGFβ, IDO, COX-2, adenosine, and arginase, and presence of regulatory T-cells and myeloid-derived suppressor cells (19, 20)], and the physical barriers that trap immune cells in the stroma [also called immune exclusion, (21, 22)]. These factors may limit the frequency and activation of effector cells within the tumor (5, 23). Moreover, a dysfunctional T-cell state characterized by the abundance of intra-tumoral PD-1^hi^ T-cells hampered TCB activity *ex vivo* (24), providing an additional primary resistance mechanism affecting TCB activity.

TCB-induced T-cell activation is has been shown to upregulate PD-1 expression on T-cells and induce PD-L1 expression on tumor cells (IFNγ driven) (8, 9, 25–29). This may lead to adaptive immune resistance mechanisms related to the TCB mode of action, similar to what has been described for checkpoint inhibition (30, 31). The same studies provided pre-clinical evidence that blockade of the PD-1/PD-L1 axis restores TCB activity *in vitro* and *in vivo* and provided the rationale for combining TCBs with therapeutic strategies targeting T-cell dysfunction in the clinic to potentiate the activity of TCBs (13, 32). These studies led to several Phase 1 trials evaluating T-cell engaging bispecific antibodies in combination with checkpoint inhibitors, particularly anti-PD-1/PD-L1 antibodies (6, 7, 11).

We have previously described the so-called 2:1 TCBs that carry two tumor antigen binding moieties and a single CD3 binding moiety in an IgG-based format (33, 34). This 2:1 format shows advantageous properties over classical 1:1 TCBs (9). In the current study, we deepen the understanding of TCB mode of action by characterizing molecular and cellular features of immune cells and tumors following TCB treatment *in vivo* in humanized mice and syngeneic tumor models, and provide additional evidence that combination with checkpoint inhibitors of the PD-1/PD-L1 axis improves TCB activity. We demonstrate that combination treatment increases the frequency of total intra-tumor T-cells, and identify the CXCL10-CXCR3 pathway as one of the potential mechanisms mediating such increase. We also show that combination treatment lowers the intra-tumor frequency of putatively exhausted T-cells. Together, the study corroborates the relevance of blocking the PD-1/PD-L1 axis to improve TCB activity and highlights the importance of exploring additional combinations that enable generation of T-cells maintaining the optimal functional status.

## Materials and Methods

### Therapeutic Antibodies

The human carcinoembryonic antigen TCB (CEA-TCB; cibisatamab) monoclonal antibody was generated as described previously (8). A murine surrogate of CEA-TCB (muCEA-TCB) was generated for studies in fully immunocompetent mice on a fully silent murine IgG_1_ backbone. MuCEA-TCB antibody was generated using an anti-CEA binder that binds to a partially overlapping (but not competing) epitope to the human CEA binder include in CEA-TCB antibody and contains the murine-specific anti-CD3 binder (clone 2C11). The potency of muCEA-TCB is about 10-fold lower than the potency of human CEA-TCB, attributed to the lower activity of the murine anti-CD3 antibody clone and putatively to the lower cytotoxic activity of murine splenocytes in *ex vivo* killing assays used to profile the activity of the surrogate molecule (data not shown). The anti-PD-L1 monoclonal antibody used in the humanized NOG mouse studies is the clone YW243.55.S70 on a muIgG1 DAPG backbone. The anti-PD-L1 monoclonal antibody used in the immunocompetent mouse studies was mIgG1 anti-PD-L1 antibody (clone 6E11), which reacts to human and murine PD-L1) (35).

### Cell Lines

The MKN-45 human gastric adenocarcinoma cell line used in humanized NOG mouse studies was purchased from DSMZ (Braunschweig, Germany; Cat No.: ACC 409). The cells were cultured in DMEM containing 10% FCS and 1% Glutamine and split 1:3 to 1:5 every 2–4 days. The HPAF-II pancreatic adenocarcinoma cell line that was also used in humanized NSG mouse studies was purchased from the American Type Culture Collection (Manassas, VA 20110 USA; Cat No.: CRL-1997). The MC38-hCEA for use in the immunocompetent human CEA transgenic mouse (huCEA Tg mice) study were derived from a mouse colon adenocarcinoma and engineered to express human CEA, obtained from Beckmann research institute of City of Hope (Duarte, CA, USA) (36). The WSU-DLCL2 human diffuse large cell B cell lymphoma (DLBCL) cell line used in the humanized mice studies with CD20-TCB were obtained from the European Collection of Cell Culture. MV3 is a human melanoma cell line, that was established by Ruiter DJ (Department of Pathology, University Hospital Nijmegen, Netherlands) (37). The cells were cultured in DMEM, containing 10% FCS and 1% GlutaMAX and split 1:3 to 1:6 every 3–4 days. Skov3 (ATCC, HTB-77) is a human ovary adenocarcinoma cell line. The cells were cultured in RPMI containing 10% FCS and 1% Glutamine and split 1:4 to 1:8 every 4 days. HT-29 (ATCC, HTB-38) is a human, female Caucasian colon adenocarcinoma cell line. The cells were cultured in McCoy’s 5A +10% FCS and 2nM GluMax and split 1:3 to 1:8 every 2–4 days. LS174T (ATCC, CL-188) is a human colon carcinoma cell line. The cells were cultured in in DMEM containing 10% FCS and 1% Glutamine and split 1:3 to 1:5 every 2–4 days.

### Mouse Models

All mice were maintained under specific-pathogen-free condition with daily cycles of 12-h light/12-h darkness according to international (Federation of European Laboratory Animal Science Associations) and national [Gesellschaft für Versuchstierkunde/Society of Laboratory Animal Science (GV-Solas) and Tierschutzgesetz (TierSchG)] guidelines. The study protocol was reviewed and approved by the local government (license ZH193/2014). Animals were maintained for 1 week after arrival to get accustomed to the new environment and for observation. Daily continuous health monitoring was conducted.

Hematopoietic stem cell humanized mice were generated in house. Briefly, 4–5-week-old female NOG (NOD.Cg-*Prkdc^scid^ Il2rg^tm1Sug^*) mice (Taconic, Cologne, Germany) or NOD scid gamma (NSG) mice (Jackson Laboratory, Sacramento, CA USA) were injected i.p. with 15 mg/kg Busulfan (Busilvex, Pierre Fabre Limited) in a total volume of 200 μl. Twenty-four hours later, mice were injected intravenously (i.v.) with 1 × 10^5^ CD34+ cord blood cells (STEMCELL Technologies Inc, Grenoble, France). Fifteen weeks after cell injection, mice were bled and screened for successful humanization by flow cytometry. The generation of these mice will be reported in detail elsewhere.

Immunocompetent human CEA transgenic (huCEA Tg) C57BL/6J mice were obtained under license agreement from Beckmann research institute of City of Hope (36). Double transgenic CEA424-SV40Tag x CEACAM5 Tg mice were obtained under license agreement from LIFE-Center of “Klinikum der Universtität München” (Prof. Dr. Wolgang Zimmermann) (38, 39). Both strains were bred by Charles River Laboratories (Lyon, France).

#### Subcutaneous Tumor Cell Inoculation

MKN-45 cells, HPAF-II cells and WSU-DLCL2 cells were cultured in RPMI containing 10% FCS (PAA Laboratories, Pasching, Austria) and 1% Glutamax (Gibco, Zug, Switzerland) at 37°C in a water-saturated atmosphere at 5% CO_2_. Afterwards, 1 × 10^6^ cells of MKN-45 or HPAF-II cells (1.5 × 10^6^ for WSU-DLCL2 cells) were injected s.c. using a 1:1 mixture of RPMI medium and Matrigel in a total volume of 100 μl.

MC38-huCEA cells were maintained in RPMI medium supplemented with 10% FCS, 500 μg/ml Geneticin (G418, Gibco). Mice were injected s.c. with 0.5 × 10^6^ cells using RPMI medium and Matrigel (1:1) in a total volume of 100 μl.

#### Therapeutic Antibody Treatment

All mice were injected i.v. or i.p. with 200 µl of the appropriate solution. The mice in the vehicle group were injected i.v. with Histidine buffer (20 mM Histidine, 140 mM NaCl, pH 6.0) and the treatment group with the antibody diluted with Histidine buffer to a volume of 200 µl.

#### Tumor Volume Measurement

Tumor volume (½ [length × width^2^]) was measured 3 times per week by caliper.

#### Necropsy

At study termination, mice were bled under anesthesia (retro-orbital) and sacrificed. Fresh blood was collected in Heparin tubes. Tumors were surgically removed from all animals and cut into three pieces. One part was snap-frozen in liquid nitrogen for RNA sequencing analysis and multiplex cytokine/chemokine analysis, one part was fixed overnight in 4% paraformaldehyde for histological analysis, and one part stored in PBS for flow cytometric analysis.

### Whole Body SPECT/CT Imaging Technique

CEA-TCB and untargeted TCB (DP47-TCB) antibodies were conjugated with 2-(4-isothiocyanatobenzyl)-1,4,7,10-tetraazacyclododecane-1,4,7,10-tetraacetic acid (p-SCN-Bz-DOTA; Macrocyclic, Plano, TX, USA) and radiolabeled with ^111^In and ^177^Lu, respectively, as described previously (40, 41). Biological and chemical analyses were performed to confirm the integrity of the radiolabeled antibodies.

Female, CD34+ human hematopoietic stem cell engrafted NOD scid gamma (NSG) mice (Jackson Laboratories, Sacramento, CA USA), age ~20 weeks were injected s.c. near the flank of one side with 1 × 10^6^ MKN-45 cells in simple RPMI medium mixed with growth factor reduced Matrigel (1:1 ratio) in 100 µl total injection volume. When tumors reached the target size of 150–300 mm^3^, mice were injected with 20 μg of ^111^In-CEA-TCB and 20 μg of ^177^Lu-DP47-TCB.

Animals were imaged with standards of ~50 µCi of each pure isotope in an Eppendorf tube placed in the field-of-view underneath the head for spillover coefficient estimation and quantification quality control. At time points of 4, 24, 72, and 120 h post-injection, whole-body, dual-isotope, energy-windowed SPECT scans were acquired, followed by CT for anatomical reference. SPECT acquisition was conducted using energy windows of 162.7–179.9 KeV and 233.1–257.6 KeV for ^111^In and 107.2–118.5 KeV and 198.0–218.8 KeV for ^177^Lu. Images were reconstructed, converted to units of µCi, co-registered to corresponding CT images, corrected for crosstalk, and then analyzed using Region of Interest (ROI) based quantification.

### Flow Cytometry

Fresh mouse heparin blood (200 μl) was lyzed using the BD Pharm Lyse™ lysing buffer (BD Biosciences, Eysins, Switzerland; Cat No.: 555899) according to manufacturer instructions. Tumors were harvested in sterile PBS and dissociated using the gentleMACS™ system (Miltenyi Biotec, Solothurn, Switzerland). Briefly, tumors were added to C-tubes (Miltenyi Biotec) in a total volume of 5 ml RPMI medium containing Collagenase D solution (Roche, diluted in PBS, final concentration: 1 mg/ml), Dispase II solution (Roche, diluted in PBS, final concentration: 0.64 mg/ml) and DNAse I solution (Roche, diluted in PBS, final concentration: 0.025 mg/ml). After running the tumor program #1, the suspension was incubated for 30 min at 37°C followed by tumor program #2. Cell suspensions were filtered using a BD Falcon™ cell strainer nylon filter (70 μm) and washed twice in FACS buffer (Dulbecco’s PBS without Ca^2+^ and without Mg^2+^, supplemented with 2% FCS and 2 mM EDTA).

Cell suspensions were stained with LIVE/DEAD^®^ Fixable Blue Dead Cell Stain Kit (Life Technologies/ThermoFisher Scientific, Basel, Switzerland) to exclude dead cells according to manufacturer instructions. Afterwards, cells were stained in FACS buffer with anti-human CD45 (Clone: HI30), CD8 (Clone: SK1), CD3 (Clone: UCHT1 or OKT3), CD4 (Clone: OKT4), PD-1 (Clone: EH12.2H7), 4-1BB (Clone: 4B4-1), Ki-67 (Clone: Ki-67), and granzyme B (GZMB) (Clone: GB11), or anti-mouse CD45 (Clone:30-F11), CD3 (Clone: H57-597), CD8 (Clone: YTS156.7.7 or 53-6.7), CD4 (Clone: GK1.5), CXCR3 (Clone: CXCR3-173), FoxP3 (Clone: MF-14), CD62L (Clone: LMEL-14), CD44 (Clone: IM7), PD-1 (Clone: RMP1-30), Tim-3 (Clone RMT3-23), Lag-3 (Clone: C9B7W). All antibodies were obtained from BioLegend/Lucerna-Chem, Luzern, Switzerland except for CXCR3 which was obtained from BD Biosciences. For intracellular staining of Ki-67 and GZMB, first surface staining was performed followed by washing and fixation/permeabilization using BD Cytofix/Cytoperm™ (BD Biosciences) before incubation with antibodies for intracellular staining. Final cell suspension was washed and acquired using a BD LSRFortessa™ cell analyzer (BD Biosciences). Manuel gating was carried out using Flowjo. Living CD45+ tumor-infiltrating immune cells were further analyzed to define specific immune cell subsets and their activation and differentiation status.

### Histological Analysis

Briefly, tumor tissue was fixed in 4% paraformaldehyde overnight and embedded in paraffin. Sections (4 μm) were cut using a microtome (Leica) and mounted on glass slides. Samples were de-paraffinized and heat antigen retrieval was performed prior to immunostaining with antibodies specific for human CEA (Roche in house), human CD3 (Abcam, Cat No.: ab5690), and human PD-L1 (Ventana, Cat No.: 790-4905). Sections were counterstained with hematoxylin (Sigma Aldrich) and slides were scanned using Olympus VS120-L100 Virtual Slide Microscope. Quantification of percentage of positive stained tumor area for PD-L1 or CEA was performed in whole scans with Definiens software. Raw data was transferred to GraphPad software for analysis of significance. A total of five mice per treatment group was evaluated.

### Cytokine/Chemokine Analyses

Cytokine/chemokine analyses from tumors of humanized mice were conducted using the Bio-Plex Pro™ Human Chemokine Panel, 40-Plex (Bio-Rad Laboratories AG, Cressier, Switzerland, Cat No.: 171AK99MR2). Small tumor fragments were snap frozen and whole protein was isolated in the presence of EASYpack Protease Inhibitor Cocktail (Roche; ref 5892970001) using the Precellys^®^24 Homogenizer and Bio-Plex^®^ Cell Lysis Buffer following manufacturer instructions. Whole protein content was measured with BCA™ Protein Assay Kit (Thermo Scientific) before cytokine measurement was performed.

### RNA Sequencing Analysis

High molecular weight RNA (>200 base pairs) was extracted and RNAseq libraries were generated and sequenced using the TruSeq^®^ Stranded mRNA kit (Illumina^®^) as per manufacturer’s instructions at Expression Analysis Inc.

Reads for each sample were processed using the following steps: First, reads were aligned to the human and mouse transcriptome (based on Ensembl v60) using Bowtie2 (42) with sensitive settings. In a second step, yet unmapped reads were aligned to the Human and mouse genome (hg19), and both mappings to genome and transcriptome were combined using in-house software. Reads mapped to both transcriptomes at the same time were discarded from further analysis. Raw counts were used to create an R DGEList object [edgeR version 3.24.3 (43)].

Normalization factors were calculated using the calcNormFactors function. Genes were normalized by Trimmed Mean of M-values (TMM), and were subjected to DE analyses using the voom and lmFit functions in the limma package [version 3.38.3; (44)]. Gene set enrichment analysis was conducted using the fgsea R package [Version 1.8.0; (45)] with minSize=55, maxSize=500 an nperm=100000. Genes were ranked by the corresponding log2 fold-change and GO gene sets (C5) from MsigDB signaling database (46) were used.

### Upregulation of PD-1/PD-L1 *In Vitro*

Surface expression of PD-1 on CD4+ or CD8+ T-cells and PD-L1 on surviving tumor cells was assessed after a classical tumor cell lysis assay. Briefly, peripheral blood mononuclear cells (PBMCs) from healthy volunteers were isolated with standard techniques. MKN-45 target T-cells were plated at a density of 1.4 × 10^6^ cells/well in flat-bottom 24-well plates 1 day before the assay. CEA-TCB or untargeted TCB were then added at concentrations ranging from 6.4 pM to 100 nM and PBMCs were added to obtain a final Effector : Target (E:T) ratio of 10:1 in a final volume of 1.1 ml per well. All experiments were performed in triplicate.

Target T-cell killing was assessed after 24 and 48 h of incubation by quantification of released lactate dehydrogenase (LDH) using an LDH detection kit (Roche Applied Science, Cat No.:11 644 793 001) according to manufacturers’ instructions. Plates were read on a Spectramax ELISA reader and EC50 values were calculated.

Surviving tumor cells were detached Cell Dissociation Buffer (Gibco) and transferred into fresh 96-round-bottom well plates with the remaining PBMCs. FACS analyses were conducted on a BD Biosciences Fortessa system using fluorescently labeled antibodies specific for CD4 (BioLegend, Cat No.: 300532; BD Biosciences, Cat No.: 552838), CD8 (BioLegend, Cat No.: 301014; BD Biosciences, Cat No.: 563256), PD-1 (BioLegend, Cat No.: 329920), PD-L1 (BioLegend, Cat No.: 329708), and EpCAM (Miltenyi Biotech, Cat No.: 130-091-253).

To determine the impact of IFNγ on the PD-L1 expression on tumor cell lines, tumor cell lines were incubated for 48 h with 100 ng/ml IFNγ, and PD-L1 expression levels were determined by flow cytometry. Briefly, adherent cells were harvested using trypsin-EDTA (Life Technologies), washed with cell culture medium once and re-suspended with the respective cell culture medium with 100 ng/ml human IFNγ (PeproTech, 300-02). As reference, cells were plated in medium without IFNγ. After 48 h of incubation at 37°C, 5% CO2 in a humidified incubator, cells were harvested using cell dissociation buffer, washed with FACS buffer (PBS, 0.1% BSA) and stained using 40 µl FACS buffer containing 5 µl anti-PD-L1 antibody (BioLegend 329706) or 10 µl of the isotype control (mouse IgG2b, BD 556437). After 30 min at 4°C, cells were washed twice with FACS buffer and re-suspended in 200 µl FACS buffer containing 2% PFA to fix the cells for 20 min at RT in the dark. Finally, cells were analyzed using a BD FACS Fortessa, equipped with FACS Diva software.

In some experiments, anti- IFNγ blocking antibodies were added to the co-cultures to assess the impact on PD-L1 expression. Briefly, PBMCs from a healthy donor (obtained from Lonza) were co-cultured with MKN-45 or LS174T target cells. 25,000 target cells and 250,000 PBMCs were plated per well in flat-bottom 96-well plates, alternatively 25,000 target cells were plated without PBMCs. The anti-IFNγ antibody (InVivoMAb, Cat No.: BE0235) was added and cells were incubated for 30 min in a humidified incubator at 37°C and 5% CO_2_. After 30 min the CEA-TCB, an untargeted TCB or media were added. The final concentration of the anti-IFNγ antibody was 5 ug/ml and that of the TCBs ranged from 100 nM to 6.4 pM in a final volume of 200 µl.

After 24, 48, or 72 h the PBMCs and tumor cells (adherent cells were detached using Cell Dissociation Buffer from Gibco) were transferred into fresh 96-well round-bottom plates. FACS analysis was conducted on a BD Biosciences Fortessa system using fluorescently labeled antibodies specific for CD4 (BioLegend Cat No.: 300532), CD8 (Biolegend Cat No.: 344704), CD25 (Biolegend Cat No.: 302614), CD69 (BioLegend Cat No.: 310934), PD-L1 (Biolegend Cat No.: 329706) and EpCAM (Miltenyi Biotec 130-091-254). Viable and dead cells were discriminated using Zombie Aqua Fixable Viability Kit (BioLegend Cat No.: 423102).

### Statistical Considerations

Statistics are described in the legends.

Tumor growth inhibition values were calculated according to the equation:

$$TGI:\frac{100-Av\left(T\_treatment^{\left[day\;x\right]}-T\_treatment^{\left[baseline\right]}\right)}{Av\left(T\_vehicle^{\left[day\;x\right]}-T\_vehicle^{\left[baseline\right]}\right)}\times 100$$

### TCB-Treatment Score

Based on the Principal Component Analysis (PCA) of the ImmunoPD data, markers of PC1 (MIP-a, CXCL10, CXL13) and PC2 (CD8+ T-cells and PD-1+on CD8+ T-cells) were taken into account. The TCB treatment score summarizes the relative expression levels of MIPa, CXCL10 and CXCL13 as well as the levels of intra-tumor CD8+ T-cells and PD-1 expressing CD8+ T-cells. For a robust marker development, the estimation was based on the quintiles of the corresponding distributions of the markers in the present cohort. For every sample, depending on the relative expression of the particular marker, the marker got a discrete point ranging from 0 to 3: 0 for relative expression from the first quartile, up to 3 for the values from the last quartile of the corresponding distribution of marker values in the cohort. By applying this procedure, for each sample, an inhibitory receptor score in the range of 0 ≤ inhibitory receptor ≤ 15 by summing up the points for the five corresponding markers was obtained. Finally, each inhibitory receptor score was normalized by 15. To compare the TCB-treatment score, the Wilcox test using JMP12 (JMP, Version 12; SAS Institute Inc., 1989–2007) have been applied.

## Results

### Tumor Targeting of CEA-TCB

Dual-isotope SPECT/CT imaging of hematopoietic stem cell humanized NSG mice (HSC-NSG) bearing a human gastric adenocarcinoma xenograft tumor (MKN-45, displaying high CEA expression) showed tumor targeting and uptake of ^111^In-CEA-TCB apparent at 4 h post-antibody injection, which continued to increase up to 120 h post single antibody injection (**Supplementary Figure 1A**). At the same time, the signal of ^111^In-CEA-TCB in the blood stream and other organs gradually decreased. There was negligible tumor uptake of the ^177^Lu-Untargeted-TCB injected simultaneously in the same mice. Quantitative analyses of the images revealed at least five-fold greater tumor uptake of ^111^In-CEA-TCB than ^177^Lu-Untargeted-TCB at all time-points. The amount of ^111^In-CEA-TCB and ^177^Lu-Untargeted-TCB in the spleen was identical, whereas the amount of ^177^Lu-Untargeted-TCB in the blood pool was greater than that of ^111^In-CEA-TCB (**Supplementary Figure 1B**). Together, imaging data indicated a tumor-specific targeting and accumulation of CEA-TCB over time along with clearance from the blood stream and other organs not expressing CEA.

### CEA-TCB Treatment Reduces Tumor Growth and Generates a Highly Inflamed Tumor Microenvironment

**Figure 1:**
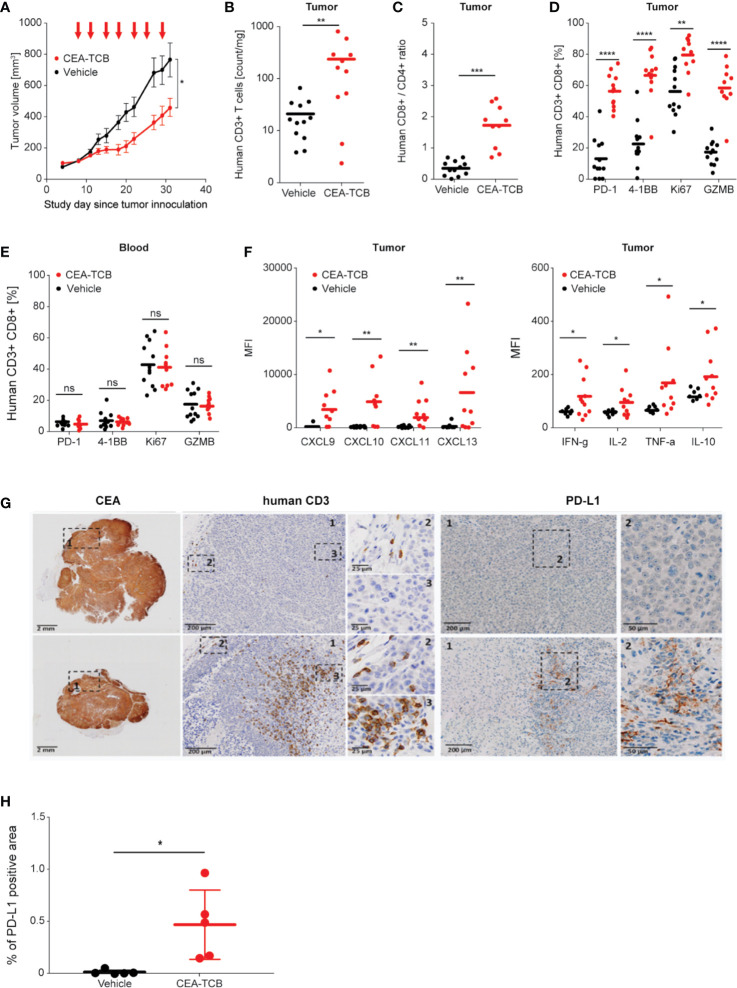
Treatment with CEA-TCB induces tumor growth inhibition and leads to increased frequency of tumor-infiltrating human T-cells and a tumor-specific T-cell activation in MKN-45-bearing hematopoietic stem cell humanized mice. Hematopoietic stem cell humanized NOG mice were inoculated subcutaneously with 1 × 10^6^ MKN-45 cells and treated with either buffer (vehicle; n=12) or with 2.5 mg/kg i.v. of CEA-TCB (n=12) twice weekly starting with a tumor volume of ~150 mm^3^ (Day 8). At termination (Day 32), blood and tumors were harvested for subsequent flow cytometry, histological and cytokine analysis (ImmunoPD data). **(A)** Tumor growth kinetics revealed a tumor growth inhibition (TGI) of 62%. Arrows indicate treatments (seven in total). **(B–E)** Flow cytometry analysis of tumor and blood in vehicle- and CEA-TCB-treated animals showing the frequency of tumor-infiltrating T-cells **(B)** and ratio of CD8+ to CD4+ T-cells in the tumor tissue **(C)**, the expression of activation markers in tumor **(D)** and blood **(E)**. **(F)** Cytokine/chemokine expression in tumor lysates. **(G)** Representative histological staining for human CEA, CD3, and PD-L1 on paraformaldehyde fixed tumor samples from vehicle (upper row) and CEA-TCB-treated animals (lower row). **(H)** Quantification of PD-L1 staining by IHC. **(A)** Data are mean ± SEM; **(B–F, H)** solid bars represent mean values; p-values are two-tailed unpaired t-test; *p < 0.05; **p < 0.01; ***p < 0.001; ****p<0.0001.

Treatment of hematopoietic stem cell humanized NOG mice (HSC-NOG) bearing human MKN-45 tumor cells with CEA-TCB 2.5 mg/kg twice weekly reduced tumor growth by 62% compared with vehicle treated controls (**Figure 1A**). Flow cytometry analysis of tumors harvested after seven consecutive treatments showed that CEA-TCB treatment induced >10-fold increase in intra-tumor T-cells and >3-fold increase in the intra-tumor CD8/CD4 T-cell ratio (**Figures 1B, C, G**). Tumor-infiltrating T-cells demonstrated an activated phenotype as detected by increased expression of 4-1BB (CD137), an activation-induced T-cell costimulatory molecule (47), and PD-1, a hallmark of T-cell activation in this context (**Figure 1D**). Tumor-infiltrating T-cells also displayed a cytotoxic potential, exemplified by increased frequency of granzyme B (GZMB)-expressing cells, and proliferation, as evidenced by increased frequency of the Ki67 positive cells (**Figure 1D**). Treatment with CEA-TCB triggered secretion of pro-inflammatory cytokines (IFNγ, TNFα, IL-2) and several chemotactic molecules (CXCL9, CXCL10, CXCL11, CXCL13) indicating the generation of a highly inflamed tumor microenvironment (**Figure 1F**). CEA-TCB treatment also triggered upregulation of PD-L1 expression in tumors (**Figures 1G, H**). There were no changes related to T-cell activation or counts in the peripheral blood upon CEA-TCB treatment, further indicating that CEA-TCB activity is restricted to areas of CEA expression, such as in tumors (**Figure 1E**). The activity of CEA-TCB was additionally assessed in a CEA-expressing pancreatic tumor models (HPAF-II) in humanized NSG mice resulting in 72% of tumor growth inhibition, and confirmed the previous observations related to TCB mode of action consisting of strong increase of intra-tumor T-cells displaying an activated phenotype and increase of CD8/CD4 T-cell ratio (**Supplementary Figure 2**). The activity of CEA-TCB was further assessed in a genetically modified CEA424-SV40 TAg transgenic model, crossed with human CEACAM5 transgenic mice that spontaneously develop gastric tumors in the pyloric region (Steinhoff N et al., in preparation). CEA-TCB treatment led to a statistically significant reduction of CEA positive tumor area accompanied by a trend towards the increase of intra-tumor T cell infiltration and improvement survival (**Supplemental Figures 3A–D**).

**Figure 2:**
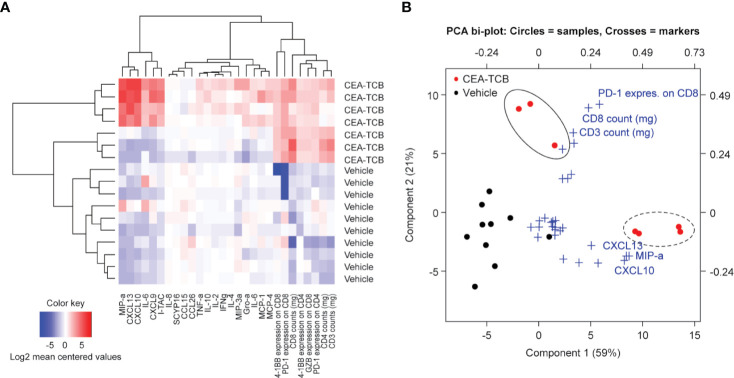
ImmunoPD data defines CEA-TCB treatment cluster. **(A)** Heatmap of ImmunoPD data from the experiment in **Figure 1**. **(B)** Principal component analysis (PCA) of the ImmunoPD data reveals a distinct cluster for the CEA-TCB-treated mice. The CEA-TCB samples are defined by two groups: One group (dashed circle) is represented by samples having a high T-cell infiltration and a high expression of chemokines (*CXCL10*, *CXCL13*, and *MIPα*) and a second group (solid circles) is represented by samples having also a high T-cell infiltration but high exhaustion state and less expression of chemokines. Crosses showing the ImmunoPD marker and its impact on the Component 1 and 2.

The heatmap generated by combining cell surface and secreted T-cell activation markers (generated by flow cytometry and multiplex analysis from the MKN-45 experiment; **Figures 1A–G**) confirmed the clear separation of the CEA-TCB-treated animals and controls. CEA-TCB-treated tumors displayed a clear upregulation of several pro-inflammatory cytokines and chemokines [MIPα (CCL3), CXCL10, CXCL13, CXCL9, IL-16, and I-TAC (CXCL11)], an increase in intra-tumor CD3+, CD4+, CD8+ T-cells that express high levels of 4-1BB, PD-1, and upregulation of GZMB (**Figure 2A**). The relative Principal Component Analysis (PCA) confirmed distinct clustering of CEA-TCB-treated mice as compared to controls (**Figure 2B**), and further revealed the presence of two sub-clusters within the CEA-TCB-treated mice: one associated with high infiltration of activated T-cells (expressing high levels of PD-1, 4-1BB, and GZMB) and high expression of pro-inflammatory chemokines and cytokines (particularly CXCL10, CXCL13, and MIPα); the other associated with high infiltration of activated T-cells (expressing high levels of PD-1, 4-1BB, and GZMB) but low expression of pro-inflammatory chemokines and cytokines (CXCL10, CXCL13, and MIPα; **Figure 2A**). The correlation of these two inflamed tumor phenotypes with tumor volume or TCB activity with regards to tumor regression did not reveal a meaningful association (data not shown).

We further defined a *CEA-TCB-treatment score* (methods) with the aim to identify the parameters that most robustly characterize the CEA-TCB activity. The TCB-treatment score was generated based on the PCA of the ImmunoPD data considering top markers of Principal Component 1 (PC1) (MIP-a, CXCL10, CXCL13) and Principal Component 2 (PC2) (CD8+ T-cells and PD-1+ on CD8+ T-cells). CEA-TCB-treated tumors have a significantly higher CEA-TCB- treatment score (p=0.0044) compared to vehicle treated tumors (**Supplementary Figure 4**).

### Identification of Gene Signature Associated With CEA-TCB Treatment

**Figure 3:**
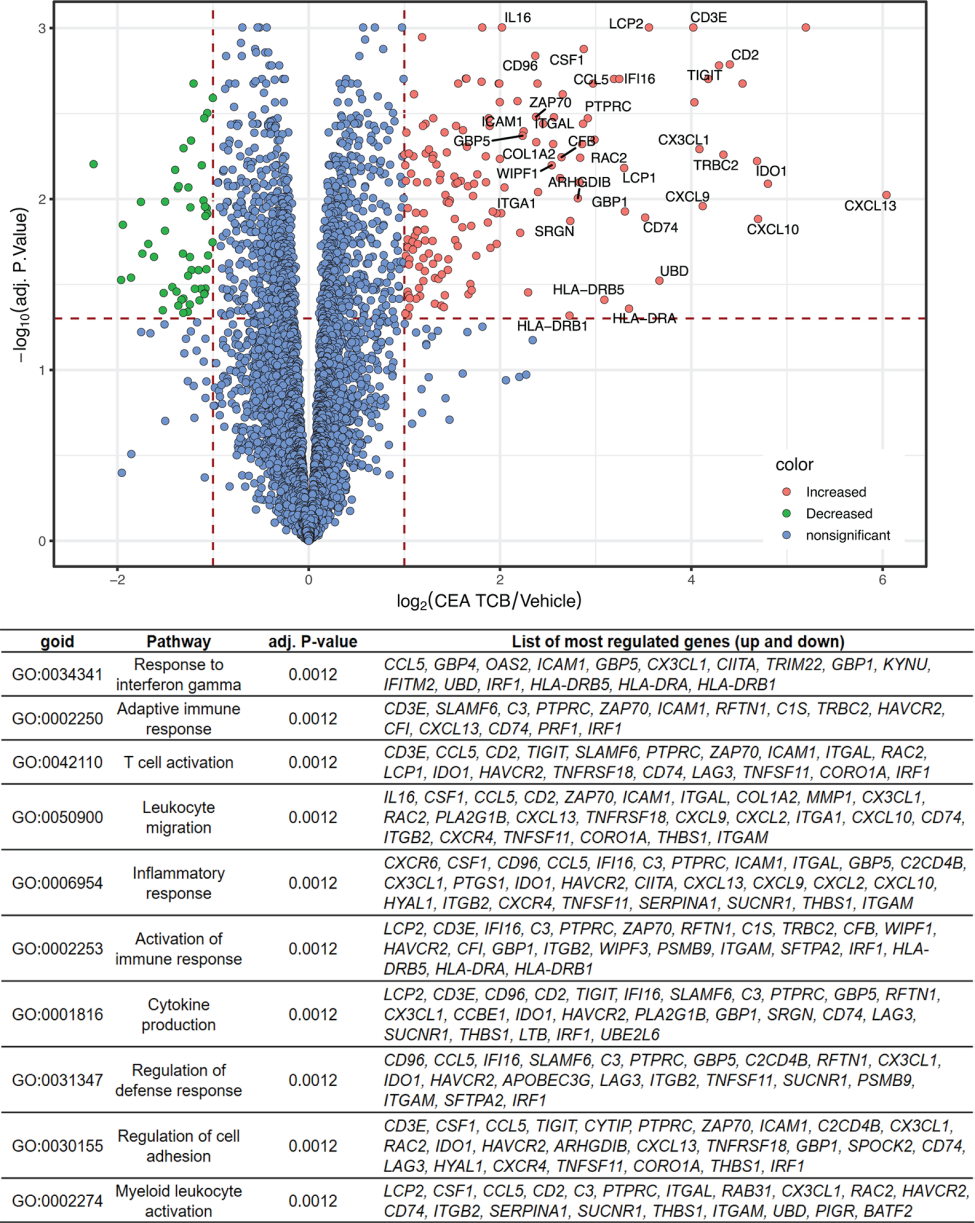
RNA sequencing data showing differentially expressed genes and Gene Ontology pathways between CEA-TCB-treated mice and controls. Tumors from the experiments in Figure 1 were harvested after seven consecutive treatments and subjected to RNA sequencing. The Volcano-plot (upper panel) displays the log2 gene expression fold change between CEA-TCB vs vehicle group (X axis) in function of the –log10 adjusted p-value using Benjamini & Hochberg correction (Y axis). Gene names are shown for genes having a log2 fold-change >2 and an adjusted p-value < 0.05. The Gene Ontology families generated considering the most deregulated genes upon CEA-TCB treatment (adj.pval < 0.05) are summarized in the table below.

To more broadly characterize the molecular parameters associated with CEA-TCB treatment we performed bulk RNA sequencing of CEA-TCB-treated (seven consecutive treatments) and untreated tumors derived from MKN-45 tumor-bearing humanized mice (experiment from **Figure 1**; GEO accession number GSE155887). Similar to the ImmunoPD analysis described above, RNA sequencing analysis revealed a clear distinction between CEA-TCB-treated and control animals, with several genes being upregulated in tumors treated with CEA-TCB as compared to controls (**Figure 3**). The list of all genes that were found to have a significantly different expression (absolute log2 fold-change > 1 and an adjusted p-value < 0.05) between CEA-TCB-treated tumors and controls is provided in **Supplementary Table 1**.

Among the top 15 upregulated genes in CEA-TCB-treated tumors there were many reflective of a strong T-cell activation, migration, immune cell response (*CXCL13, GNLY, GZMB, CXCL10, IDO1, SLA, CD2, TRBC2, TIGIT, IL2RB, CXCL9, CX3CL1, CCL4L2*, and *CD3E*). Among the top downregulated ones we found *keratin 6A* and *keratin 20* (*KRT6A* and *KRT20*) suggestive of the reduction of tumor cells as the result of TCB-mediated killing (**Figure 3** and **Supplementary Table 1**). The Gene Set Enrichment Analysis (GSEA) further enabled identification of the main biological pathways upon CEA-TCB treatment and confirmed that the main Gene Ontology families that characterize the TCB response consist of T-cell activation (*Response to interferon gamma; Adaptive Immune Response; T-cell activation; Inflammatory Response; Activation of Immune response; Cytokine secretion*) and migration (*Leukocyte Migration; Regulation of Cell Adhesion*) (**Figure 3** and **Supplementary Figure 5**). Interestingly, we also noticed upregulation of many major histocompatibility class II molecules that are known to be expressed on antigen presenting cells (*HLA-DRA, HLA-DRB5*, and *HLA-DRB1*) along with *CX3CL1* (fractalkine, a known monocyte/T-cell attractant molecule) and *CSF1* (colony stimulating factor 1, macrophage) suggestive of myeloid cell recruitment and activation at tumor sites post TCB treatment. We also observed the upregulation of PD-1 and PD-L1 transcripts following CEA-TCB treatment as compared to controls (**Supplementary Figure 6** **).**

### CEA-TCB Treatment Induces Upregulation of PD-1 and PD-L1 Expression; Combination of CEA-TCB With Anti-PD-L1 Blocking Antibody Enhances Its Efficacy in Stem Cell Humanized and Fully Immunocompetent Mice

**Figure 4:**
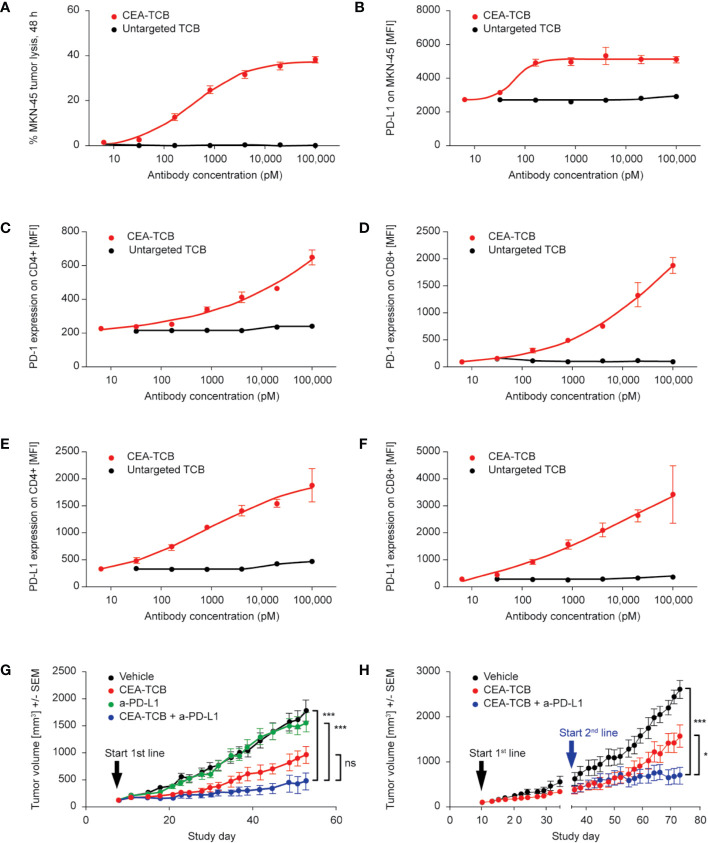
TCB-mediated cytotoxicity induces the expression of PD-1 and PD-L1; blockade of PD-1/PD-L1 axis improves the efficacy of CEA-TCB in humanized mice. **(A–F)** Example of CEA-TCB-mediated tumor cell lysis *in vitro* leading to T-cell activation with parallel upregulation of PD-1 (on both CD4+ and CD8+ T-cells) and PD-L1 (on MKN-45 tumor cells). Data are the mean and standard deviation of triplicate experiments. **(A)** Tumor cell lysis as measured by LDH release assay in a co-culture assay of human PBMC, MKN-45 tumor cells [effector:target (E:T) ratio: 10:1] in presence of increasing concentrations of either CEA-TCB or an untargeted TCB after 48 h of incubation. **(B)** Flow cytometry analysis for PD-L1 expression (MFI) on MKN-45 cells recovered after TCB-mediated killing from co-culture assays. **(C, D)** Flow cytometry analysis for PD-1 expression (MFI) on human CD4+ and CD8+ T-cells recovered after TCB-mediated killing from co-culture assays. **(E, F)** Flow cytometry analysis for PD-L1 expression (MFI) on human CD4+ and CD8+ T-cells recovered after TCB-mediated killing from co-culture assays. **(G, H)** Hematopoietic stem cell humanized NOG mice were inoculated subcutaneously with 1 × 10^6^ MKN-45 cells and treated with i.v. buffer (vehicle) twice weekly or with 2.5 mg/kg i.v. CEA-TCB twice weekly or 10 mg/kg i.v. of anti-PD-L1 once weekly, or with a combination of CEA-TCB plus anti-PD-L1 (given at the same dose and schedule as in monotherapy groups) starting with a tumor volume of ~150 mm^3^. Tumor growth kinetics are shown as mean ± SEM for all treatment groups (n=9 mice per group). **(G)** Combination treatment of CEA-TCB and anti-PD-L1 started from the beginning (Day 8; 1^st^ line treatment). **(H)** Combination treatment of CEA-TCB with anti-PD-L1 started once animals progressed to CEA-TCB monotherapy treatment (on Day 35; 2^nd^ line treatment). p-values are one-way ANOVA with Tukey’s multiple comparison correction: ns, not significant; **p < 0.01, ***p < 0.001.

Data shown in **Figures 1D, G, H** and **Supplementary Figure 6** provided evidence of PD-1 and PD-L1 upregulation on T-cells and tumors upon *in vivo* treatment with CEA-TCB. Additional evidence of the dose-dependent PD-1 upregulation on CD4 and CD8 T-cells as well as PD-L1 upregulation on tumor cells and T-cells upon CEA-TCB treatment was obtained from *in vitro* experiments (**Figures 4A–F**; **Supplementary Figure 6**). Incubation of the CEA-expressing MKN-45 target cells with human PBMC in presence of increasing concentrations of CEA-TCB led to the expected tumor cell lysis (**Figure 4A**). Flow cytometry analysis of co-cultured cells upon treatment revealed dose-dependent upregulation of PD-L1 on tumor cells (**Figure 4B**) and dose-dependent upregulation of PD-1 (**Figures 4C, D** and **Supplementary Figures 7A, B**) and PD-L1 (**Figures 4E, F** and **Supplementary Figures 7C, D**) on CD4+ and CD8+ T-cells compared with cells incubated with untargeted TCB control. IFNγ is the main mediator of PD-L1 upregulation on tumor cells (**Supplementary Figure 8A**) and is released by CEA-TCB activated T cells in co-culture with tumor cells (**Supplementary Figures 8B, C**). Treatment of tumor cells with CEA-TCB in the absence of immune cells did not lead to PD-L1 upregulation on tumor cells, further confirming the key role of activated immune cells in secreting IFNγ (**Supplementary Figures 8B, C**). Further experiments corroborated the key role of IFNγ demonstrating that blocking of IFNγ by means of neutralizing antibodies reduced the PD-L1 upregulation on tumor cells resulting from CEA-TCB-mediated T cell activation (**Supplementary Figures 8D, E**).

The upregulation of PD-1 on T-cells and PD-L1 on tumor and T-cells following *in vitro* and *in vivo* TCB treatment led us to investigate whether combining CEA-TCB with PD-L1 blocking antibody could enhance the anti-tumor efficacy of CEA-TCB. We initially investigated the activity of this combination in HSC NOG mice bearing MKN-45 tumors. Treatment with CEA-TCB and anti-PD-L1 blocking antibody improved anti-tumor activity compared with either agent alone; in addition, the onset of tumor regrowth was significantly delayed in the combination group compared with anti-PD-L1 single-agent treatment (**Figure 4G**). Although the difference of the combination treatment compared with CEA-TCB monotherapy was only close to being significant, data support a trend for improved efficacy of the combination effect. Stronger anti-tumor activity was achieved when the combination started from the beginning (e.g. from the first treatment cycle) (**Figure 4G**) compared with combination that started after progression to CEA-TCB monotherapy treatment (**Figure 4H**).

**Figure 5:**
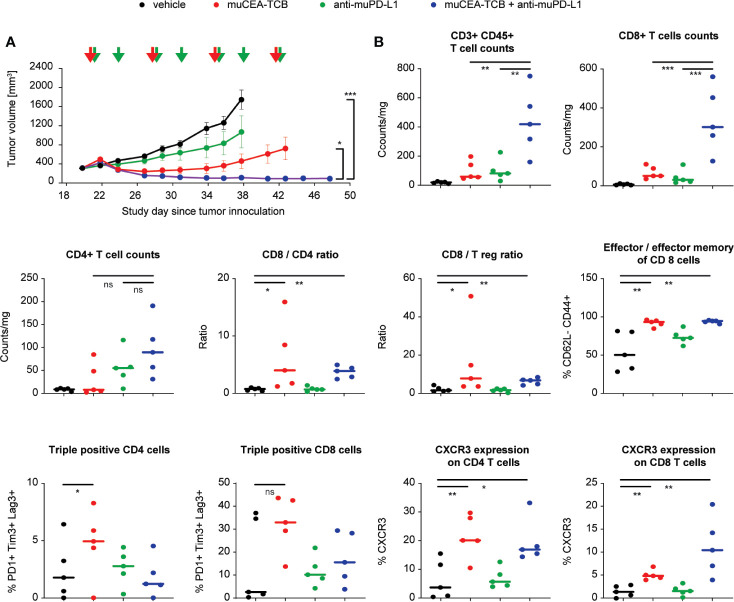
Blockade of PD-1/PD-L1 axis improves the efficacy of CEA-TCB in immunocompetent mice. **(A)** Immunocompetent human CEA transgenic (huCEA Tg) C57BL/6J mice were inoculated subcutaneously with 0.5 × 10^6^ MC-38-huCEA cells and treated with i.v. buffer (vehicle), murine surrogate of CEA-TCB (muCEA-TCB; 2.5 mg/kg i.v. once weekly), murine surrogate of anti-PD-L1 (10 mg/kg i.v. initial dose followed by 5 mg/kg i.p. twice weekly), or with a combination of muCEA-TCB and a-muPD-L1 (same dose and schedule as in monotherapy groups). Treatment started with a tumor volume of 200–400 mm^3^ (Day 20). Arrows indicate treatments. Tumor growth kinetics are shown as mean ± SEM for all treatment groups (n=16 mice per group). Combination group vs muCEA-TCB: p=0.023 and vs vehicle: p<0.001; one-way ANOVA with Tukey’s multiple comparison correction done after five treatments (Day 38). **(B)** 24 h after the third treatment (Day 29), scout mice were sacrificed, tumor tissue was obtained and analyzed for T-cell infiltration and phenotype. p-values are one-way ANOVA with Tukey’s multiple comparison correction: ns, not significant; *p < 0.05; **p < 0.01; ***p < 0.001.

The efficacy of the combination of CEA-TCB plus a PD-L1 blocking antibody was further assessed in fully immunocompetent model consisting of human CEA transgenic (huCEA Tg) C57BL/6J mice bearing a syngeneic colorectal tumor line (MC38) stably expressing human CEA (MC38-hCEA). Treatment with murine surrogate of CEA-TCB (muCEA-TCB) and of anti-PD-L1 blocking antibody (muPD-L1) led to more rapid, more pronounced and sustained tumor growth inhibition as compared to the respective monotherapy treatment groups (**Figure 5A**).

Flow cytometry analysis of treated tumors, harvested 24 h after third infusion of the molecules, revealed that, similar to studies in humanized mice, CEA-TCB monotherapy increased the frequency of intra-tumor T-cells with preferential increase of CD8 T-cells over CD4 T-cells, leading to an increased ratio of intra-tumor CD8/CD4 and CD8/Tregs (**Figure 5B**). The majority of CD8 T-cells displayed a cytotoxic effector and effector memory phenotype and a significant fraction of CD8 T-cells (35%) displayed triple expression of PD-1+Tim3+Lag3+ (putatively exhausted cells). In addition, CEA-TCB treatment increased the frequency of intra-tumor CD4 and CD8 T-cells expressing CXCR3, a key receptor regulating T-cell chemotaxis (**Figure 5B**). Interestingly, combination treatment of CEA-TCB and anti-PD-L1 blocking antibody increased the intra-tumor frequency of both CD4 and CD8 T-cells compared with monotherapies and vehicle control. This led to a similar frequency of cytotoxic effector and effector memory cells, but a lower frequency of triple positive, putatively exhausted PD-1+Tim3+Lag3+ CD8 T-cells (15% in combination vs 35% in CEA-TCB monotherapy) and higher frequency of intra-tumor CXCR3+ CD8+ T-cells (11% in combination vs 5% in CEA-TCB monotherapy) (**Figure 5B**). Together, the intra-tumor T-cell phenotype upon CEA-TCB and anti-PD-L1 combination treatment is reflective of T-cells having higher propensity for recruitment and putatively lower exhaustion status, and may reflect a higher recruitment of fresh T-cells from the peripheral blood.

### Combination With Anti-PD-L1 Also Enhances the Efficacy of CD20-TCB in Stem Cell Humanized Mice

We further demonstrated the value of combining a TCB antibody with PD-L1 blockade for hematological malignancies using CD20-TCB (glofitamab), another “2:1” format TCB targeting CD20 on B cells and CD3 on T-cells (9, 48). Glofitamab is currently under clinical development in patients with relapsed or refractory B-cell non-Hodgkin lymphoma. To better evaluate the effect of the combination, HSC NSG mice bearing an aggressive human DLBCL cell line (WSU-DLCL2) were treated with a suboptimal dose of CD20-TCB (0.15 mg/kg). This resulted in suboptimal anti-tumor activity compared to its optimal dose (9), as monotherapy or in combination with an anti-PD-L1 blocking antibody. While monotherapy treatment with CD20-TCB or anti-PD-L1 blocking antibody did not show anti-tumor efficacy, the combination treatment led to tumor growth inhibition (**Supplementary Figure 9**).

## Discussion

The current study was undertaken to expand our understanding of cellular and molecular features associated with TCB activity and to address one of the key adaptive resistance mechanisms related to TCB activity, namely PD-1/PD-L1 axis upregulation, similarly to what has been described for checkpoint inhibitors (30).

The efficacy and mode of action of single-agent CEA-TCB was evaluated in different preclinical CEA-expressing mouse tumor models. These comprised hematopoietic stem cell humanized NOG mice bearing human gastric and pancreatic tumors and immunocompetent human CEA transgenic C57BL/6J mice (hCEA Tg mice) bearing a murine colorectal cancer tumor line (MC38) or crossed with genetically modified CEA424-SV40 TAg transgenic mice that spontaneously develop gastric tumors in the pyloric region. The former represent a hyper-mutated and highly inflamed form of colorectal cancers (MSI^hi^ CRC) (49) transfected to stably express human CEA (MC38-hCEA), the latter an aggressive form of murine gastric cancer with immune desert phenotype, which is poorly responsive to cancer immunotherapy treatment (Steinhoff N et al., in preparation).

In all models, single-agent CEA-TCB slowed the growth of tumors compared with controls. Treatment of mice bearing CEA-positive tumors with CEA-TCB led to a 2 to >10-fold increase in tumor-infiltrating T-cells (depending on the tumor and mouse model). The tumor-infiltrating T-cells displayed a highly activated and proliferating phenotype, with tumors displaying a highly inflamed microenvironment as evidenced by increased levels of several pro-inflammatory cytokines and chemokines. Notably, anti-tumor efficacy along with tumor inflammation and increases of activated intra-tumoral T-cells was obtained in response to CEA-TCB treatment, even in settings with low pre-existing baseline tumor immune cell infiltration. This indicates that unlike other immunotherapies, CEA-TCB has the potential to be efficacious in patients with poor pre-existing inflammation. This is particularly relevant for patients with low frequency of pre-existing intra-tumoral CD8+ cells, who respond poorly to cancer immunotherapy (50) and particularly for the vast majority of human (CEA-expressing) colorectal cancer tumors with proficient mismatch repair (MMR) or with microsatellite stable (MSS) tumors who do not benefit from immunotherapy. Colorectal tumors with microsatellite instability (MSI) are typically more antigenic and have greater infiltration of CD8+ cells than MSI-negative tumors (51, 52).

A molecular signature of TCB treatment was identified consisting of pro-inflammatory cytokines/chemokines, and higher frequency and activation of T-cells. The signature appeared to be robust, as components of the signature were confirmed using complementary techniques: RNA expression analysis and protein expression as determined by flow cytometry and multiplex analysis. In particular, *CXCL9* and *CXCL10* were identified by both methods as the key molecules significantly upregulated by CEA-TCB treatment compared to controls. CXCL9 and CXCL10 are potent pro-inflammatory chemokines and chemoattractants for multiple immune effector cells, including NK cells, monocytes/macrophages and T-cells by binding to the CXCR3 receptor expressed on the same cells (53, 54). In line with this, CEA-TCB treatment also increased the frequency of intra-tumor CXCR3+ CD8+ and CD4+ T-cells, corroborating the relevance of the CXCL10-CXCR3 axis in mediating the attraction of T-cells leading to increase of intra-tumor T-cell infiltration upon TCB treatment (25) (and unpublished data). It will be interesting to investigate the prognostic value of the TCB-treatment score in biopsies obtained from the ongoing interventional trial of the combination of CEA-TCB (cibisatamab) and atezolizumab in previously treated metastatic colorectal adenocarcinoma patients (NCT03866239).

A clear upregulation of both PD-1 (on CD4 and CD8 T-cells) as well as PD-L1 (on tumor cells and CD4 and CD8 T-cells) was detected in response to CEA-TCB treatment, indicative of the PD-1/PD-L1 axis being one of the adaptive resistance mechanisms related to TCB activity (8, 9, 25–29). Combination of TCBs with anti-PD-L1 blocking antibody (in different tumor and mouse models and using different TCBs targeting both CEA (solid tumors) and CD20 (hematological malignancies) consistently translated into superior anti-tumor efficacy and stronger tumor growth inhibition when compared to either agent given as monotherapy. Better tumor growth inhibition was obtained when the two agents were combined simultaneously from the first treatment cycle, as compared to starting the combination when tumors progressed to CEA-TCB monotherapy. This finding is consistent with previous *in vitro* data with a CEA BiTE MEDI-565/AMG 211 that showed that T-cell killing was maximized when dual blockade of PD-1 and PD-L1 was applied early (26). Interestingly, the combination of CEA-TCB and an anti-PD-L1 blocking antibody led to increased frequency of intra-tumor CD4 and CD8 T-cells displaying a cytotoxic effector and effector memory phenotype; at the same time, the combination treatment lowered the frequency of putatively exhausted T-cells (characterized by co-expression of PD-1+Tim3+Lag3+ CD8 T-cells) and increased the frequency of T-cells having migratory capacity (characterized by CXCR3+ expression on CD8+ T-cells). Taken together, the intra-tumor T-cell phenotype upon CEA-TCB and anti-PD-L1 combination treatment is reflective of T-cells having higher propensity to migrate and putatively lower exhaustion status, and may indicate a stronger recruitment of fresh T-cells from the periphery.

These pre-clinical data support the rationale for the clinical investigation of CEA-TCB and atezolizumab, which is currently in Phase Ib (NCT03866239). Preliminary results of clinical activity indicated promising anti-tumor efficacy in patients with CEA+ solid tumors (mostly colorectal cancer) when cibisatamab was combined with the anti-PD-L1 antibody atezolizumab (55). Comparison of pre-treatment and on-treatment patient tumor biopsies (most of which came from MSS CRC patients with a non T-cell inflamed immunophenotype prior to treatment) indicated that cibisatamab and atezolizumab combination treatment led to the increase of intra-tumor proliferating T-cells, increase of PD-1+ T-cells, upregulation of PD-L1 expression on immune cells, and reduction of CEA expressing tumor cells (56, 57), corroborating pre-clinical findings presented in the current study.

In conclusion, the data of the current study expand our knowledge of the cellular and molecular features associated with TCB activity, and provide evidence that the PD-1/PD-L1 axis is one of the adaptive resistance mechanisms associated with TCB activity. This adaptive resistance mechanism can be managed by the combination of TCB with anti-PD-L1 (or anti-PD-1) blocking antibodies translating into more efficacious anti-tumor activity and prolonged control of the tumor outgrowth. However, the data also show that tumors continue to progress despite the anti-PD-L1 combination treatment, suggesting that additional mechanisms, beyond the PD-1/PD-L1 axis, contribute to tumor escape. The elucidation of such mechanisms, most likely contributed to by both tumor cells and different immune cell subsets, by using high dimensional single cell approaches for tumor analysis, will constitute an important milestone in our understanding of additional resistance mechanisms to immunotherapy and novel combination approaches for efficient tackling of the same.

## Data Availability Statement

The original contributions presented in the study are publicly available. This data can be found here: https://www.ncbi.nlm.nih.gov/geo/query/acc.cgi?acc=GSE155887.

## Ethics Statement

This study involving laboratory animals was reviewed and approved by the Institutional Animal Care and Use Committee of the Preclinical Pharmacology Department, Roche Innovation Center Zurich, Schlieren, Switzerland. This study was performed in accordance with the animal research protocols approved by the local government (Kantonale Verwaltung Veterinäramt kant. Zürich, Switzerland; license ZH193/2014). All animals were handled in accordance with the guidelines of the Federation of European Laboratory Animal Science Associations, Gesellschaft für Versuchstierkunde/Society of Laboratory Animal Science (GV-Solas) and the Tierschutzgesetz (TierSchG).

## Author Contributions

TF, MBi, and LF were in involved in the design and generation of *in vitro* data. JS and SC designed, supervised, and interpreted the *in vivo* and *ex vivo* studies. MP designed and supervised *in vivo* studies in immunocompetent mice. EB, AS, MK, ML, and NS conducted all *in vivo* and *ex vivo* experiments. VN generated all histology results. TN designed and supervised the SPECT/CT imaging study. AR supervised and analyzed the bulk RNAseq data. MBa, CK, and PU supervised the project. MBa and JS wrote the manuscript. MBa contributed to experimental design and data interpretation of all *in vitro* and *in vivo* studies. All authors reviewed the manuscript. All authors contributed to the article and approved the submitted version.

## Funding

This study and editorial support for the preparation of this manuscript were funded by F Hoffmann-La Roche Ltd.

## Conflict of Interest

The authors declare that this study received funding from F Hoffmann-La Roche Ltd. The funder had the following involvement with the study: study design, generation of the molecules tested in the study, data collection and analysis, decision to publish, and editorial support for the preparation of this manuscript. All authors are employees of F Hoffmann-La Roche Ltd. Authors JS, SC, TF, AR, MBi, LF, TN, AS, MLC, CK, PU, and MBa hold stock/stock options for F Hoffmann-La Roche Ltd and authors JS, SC, TF, VG-N, MP, TN, AS, CK, PU, and MBa hold patents related to the TCB technologies reported in the manuscript.
