# Corrigendum: Combination of T-Cell Bispecific Antibodies With PD-L1 Checkpoint Inhibition Elicits Superior Anti-Tumor Activity

**DOI:** 10.3389/fonc.2021.650149

**Published:** 2021-02-03

**Authors:** Johannes Sam, Sara Colombetti, Tanja Fauti, Andreas Roller, Marlene Biehl, Linda Fahrni, Valeria Nicolini, Mario Perro, Tapan Nayak, Esther Bommer, Anne Schoenle, Maria Karagianni, Marine Le Clech, Nathalie Steinhoff, Christian Klein, Pablo Umaña, Marina Bacac

**Affiliations:** ^1^ Roche Pharma Research & Early Development, Roche Innovation Center Zurich, Zurich, Switzerland; ^2^ Roche Pharma Research & Early Development, Roche Innovation Center Basel, Basel, Switzerland

**Keywords:** solid tumors, immunotherapy, T-cell bispecific antibody, carcinoembryonic antigen T-cell bispecific antibody, programmed death–ligand 1, combination, humanized mice

In the published article, there was an error in affiliations 1 and 2. Instead of “Roche Pharmaceutical Research & Early Development”, it should be “Roche Pharma Research & Early Development”.

In the original article, there was an error in the **Conflict of Interest** section. Author ownership interests were omitted from the Conflict of Interest.

A correction has been made to the Conflict of Interest section:

The authors declare that this study received funding from F Hoffmann-La Roche Ltd. The funder had the following involvement with the study: study design, generation of the molecules tested in the study, data collection and analysis, decision to publish, and editorial support for the preparation of this manuscript. All authors are employees of F Hoffmann-La Roche Ltd. Authors JS, SC, TF, AR, MBi, LF, TN, AS, MLC, CK, PU, and MBa hold stock/stock options for F Hoffmann-La Roche Ltd and authors JS, SC, TF, VG-N, MP, TN, AS, CK, PU, and MBa hold patents related to the TCB technologies reported in the manuscript.

The authors apologize for this error and state that this does not change the scientific conclusions of the article in any way. The original article has been updated.

