# Supplementary material for: Combination of T-Cell Bispecific Antibodies With PD-L1 Checkpoint Inhibition Elicits Superior Anti-Tumor Activity
Source: Front Oncol. 2020 Nov 30;10:575737. doi: 10.3389/fonc.2020.575737 (PMC7735156; doi:10.3389/fonc.2020.575737)
Supplement: Supplementary file 1 [file DataSheet_1.docx]

Supplementary Material

**Supplementary Figure 1. CEA-TCB administered to mice bearing human tumors targets the tumor site.**

**a**, CD34+ human hematopoetic stem cell engrafted NSG mice (n=3 per group) were inoculated with 1 × 10^6^ MKN-45 cells in the left flank and tumor allowed to grow until it reached a size of about 200 mm^3^. Tumor-bearing animals were subsequently treated simultaneously with ^111^In-labeled CEA-TCB (red signal) and ^177^Lu-labeled untargeted DP47-TCB (green signal). Progressively increasing localization of CEA-TCB to the tumor site (white arrow) was observed as early as 4 h post antibody injection. **b**, Quantitative analysis revealed a greater than five-fold increase in uptake of targeted CEA-TCB to the tumor site compared with untargeted TCB. Untargeted TCB predominantly localized to the blood stream. Data are mean +/- SD.


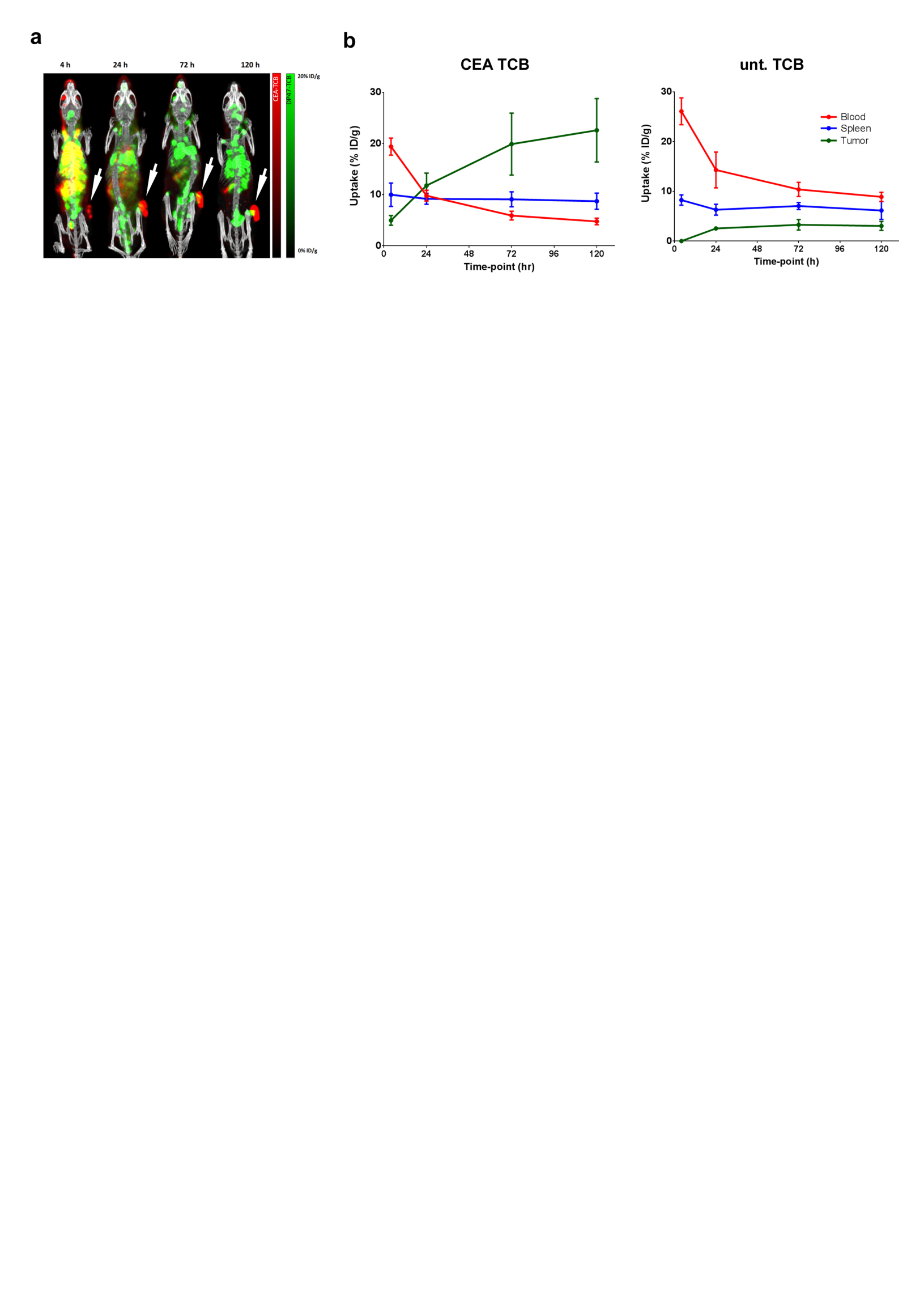


**Supplementary Figure 2. Treatment with CEA-TCB induces tumor growth inhibition and increase of frequency and activation of intra-tumor T-cells in human pancreatic (HPAF-II)- bearing humanized mice.**

Hematopoietic stem cell humanized NOG mice were inoculated subcutaneously with 1 × 10^6^ HPAF-II cells and treated with either buffer (vehicle; n=10) or with 2.5 mg/kg i.v. of CEA-TCB (n=10) twice weekly starting with a tumor volume of ~200 mm^3^ (Day 15). At termination (Day 42), five tumors were harvested for subsequent flow cytometry analysis. **a**, Tumor growth kinetics revealed a tumor growth inhibition (TGI) of 72%. Arrows indicate treatments (seven in total). **b**, Flow cytometry analysis of tumors in vehicle- and CEA-TCB-treated animals showing the frequency of tumor-infiltrating T-cell, ratio of CD8+ to CD4+ T-cells and their phenotype (4-1BB, GZBM, PD-1 and Ki-67). **c**, Representative histological staining for human CEA on paraformaldehyde fixed tumor samples from vehicle HPAF-II tumor as well as a comparison to MKN-45 in lower (upper row) and higher (lower row) magnification.


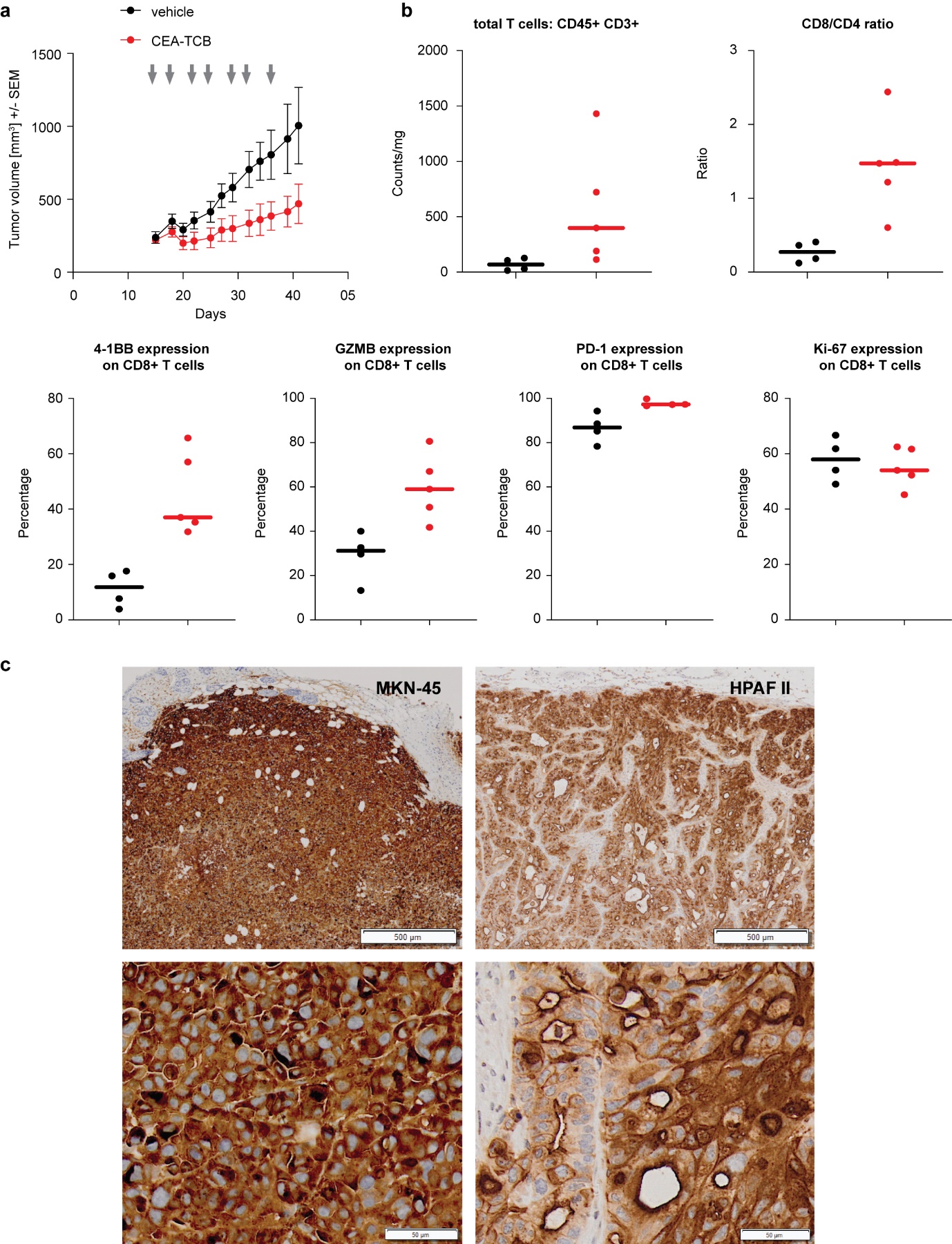


**Supplementary Figure 3. TCB-treatment in double transgenic CEA424-SV40 TAg x CEACAM5 Tg mice.**

CEA4242-SV40 TAg x CEACAM5 mice (n=5) were treated with vehicle or 2.5 mg/kg muCEA-TCB surrogate twice per week i.v. for three times. One day after the last injection, animals were sacrificed and CD3+ T cell density and CEA + tumor relative area of the pylorus was determined by histology. **a**, For survival, animals (n=3) were sacrificed once they reached termination criteria. CEA-TCB treatment increases survival of the mice in comparison to vehicle group. **b**, Amount of CEA+ tumor relative area (%) is shown for muCEA-TCB compared to vehicle. (quantification with Definiens software). Significance calculated with two-tailed unpaired t-test. ** p<0.05. Bar plots are showing mean ± SD of CEA+ relative tumor area (%) in the pyloric FFPE section. **c**, CD3+ cells (#/mm^2^) is shown in muCEA-TCB treated groups compared to vehicle (quantifications with cellular analysis of whole tissue section (n=2/group) by Definiens software). Bar plots are showing mean ± SD CD3+ cells/mm^2^ in the total pyloric FFPE section. **d**, Representative images of histology analysis of explanted tumors stained with CEA (brown) and CD3 (brown, higher magnification) treated with vehicle (left panel) and muCEA-TCB (right panel).

**
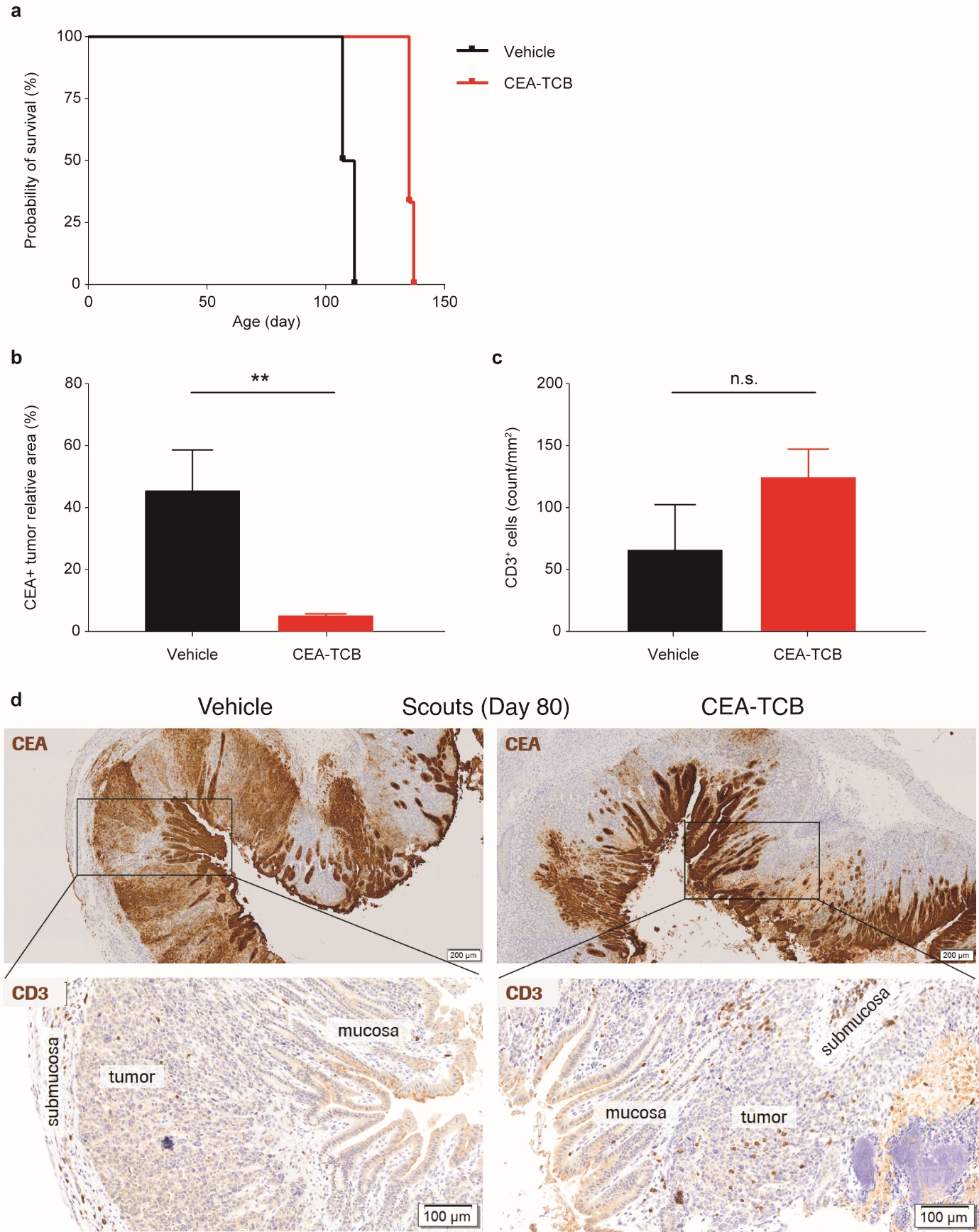
**

**Supplementary Figure 4. TCB-treatment score derived from ImmunoPD parameters.**

In order to investigate the impact of CEA-TCB treatment, a TCB-treatment score was defined (see methods). CEA-TCB treated mice have significantly higher TCB-treatment score compared to vehicle controls (mean CEA-TCB vs mean vehicle control: 0.76 vs 0.34; p=0.0044; two-sided Wilcoxon test).


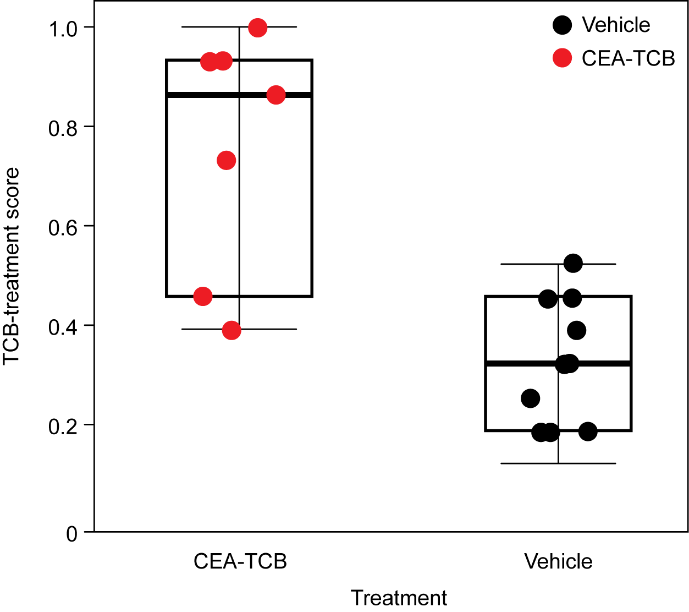


**Supplementary Figure 5. Gene set enrichment analysis (GSEA).**

Gene set enrichment analysis (GSEA, http://broadinstitute.org/gsea) was used to determine whether Gene Ontology pathways showed enrichment in CEA-TCB treated mice. Enrichment plots of the main Gene Ontology pathways shown in Figure 3 are illustrated.

**
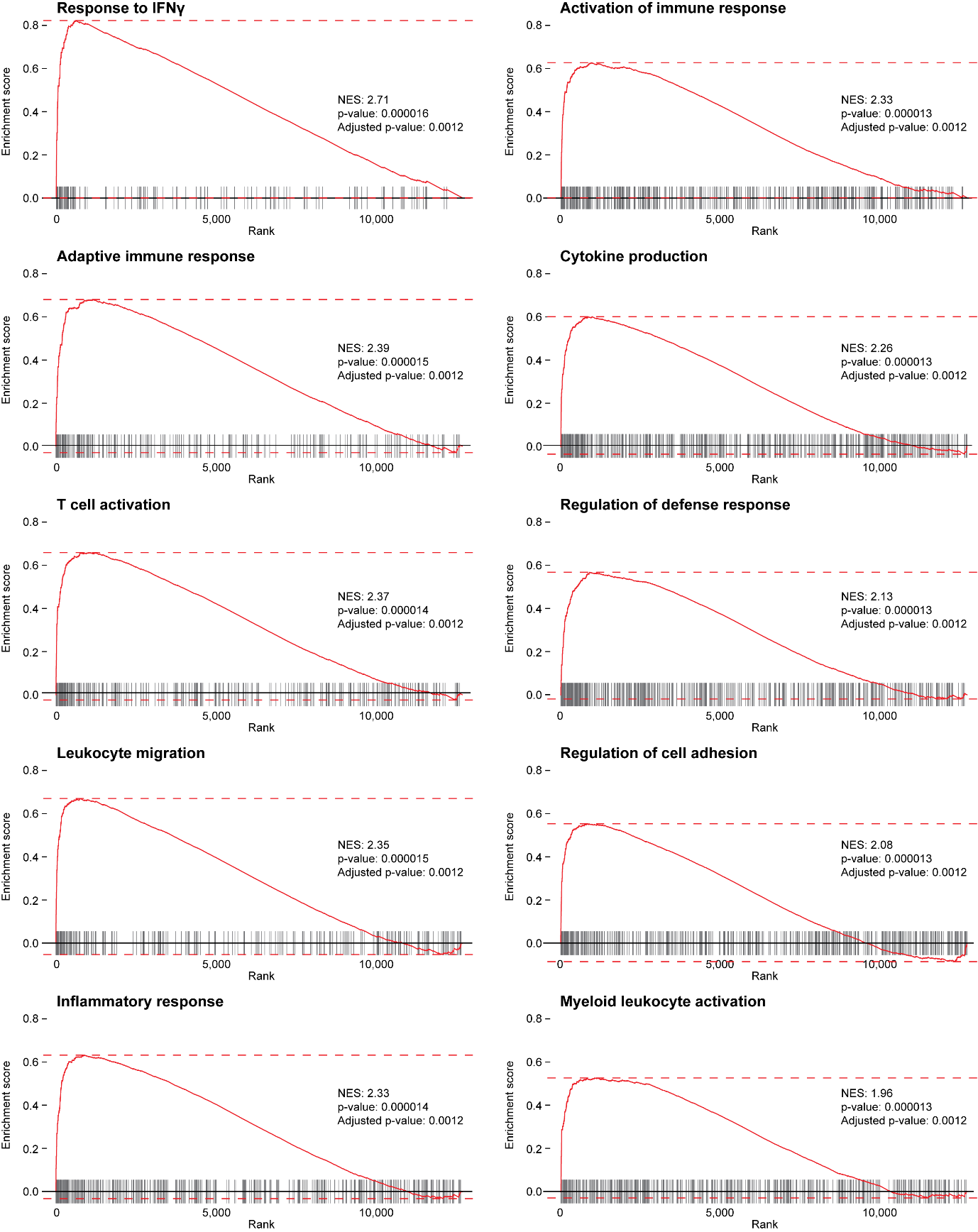
**

**Supplementary Figure 6. Log2 CPM of PD-L1 and PD-1 mRNA from bulk RNAseq.**

**
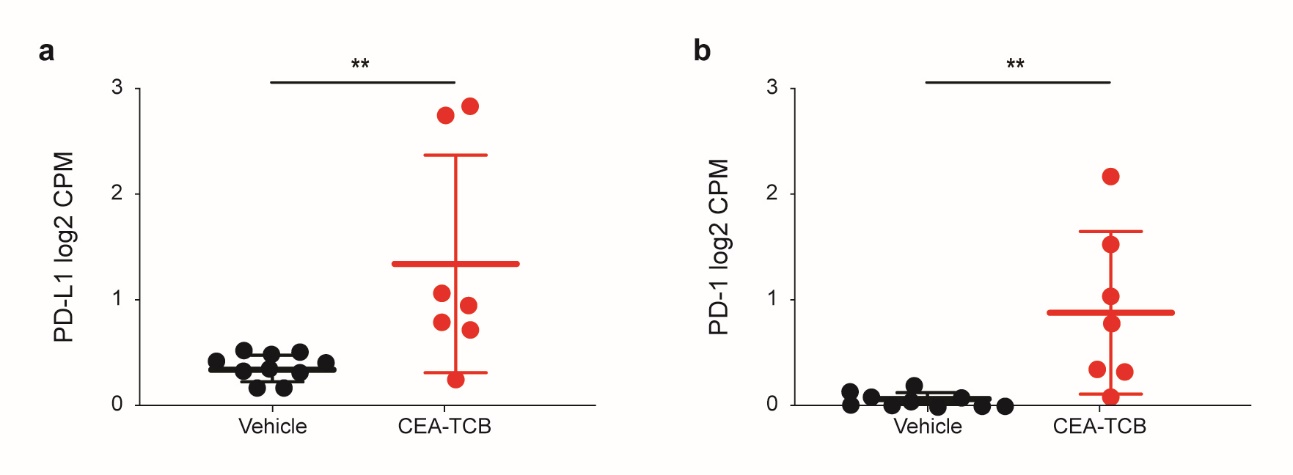
**

**Supplementary Figure 7. TCB-mediated cytotoxicity induces the expression of PD-1 and PD-L1.**

**a-d,** Percentage of upregulation of PD-1 and PD-L1 (on both CD4+ and CD8+ T-cells) *in vitro* upon CEA-TCB treatment. Data are the mean and standard deviation of triplicate experiments. **a-b**, Flow cytometry analysis for PD-1 expression (%) on human CD4+ and CD8+ T-cells recovered after TCB-mediated killing from co-culture assays. **c-d**, Flow cytometry analysis for PD-L1 expression (%) on human CD4+ and CD8+ T-cells recovered after TCB-mediated killing from co-culture assays.


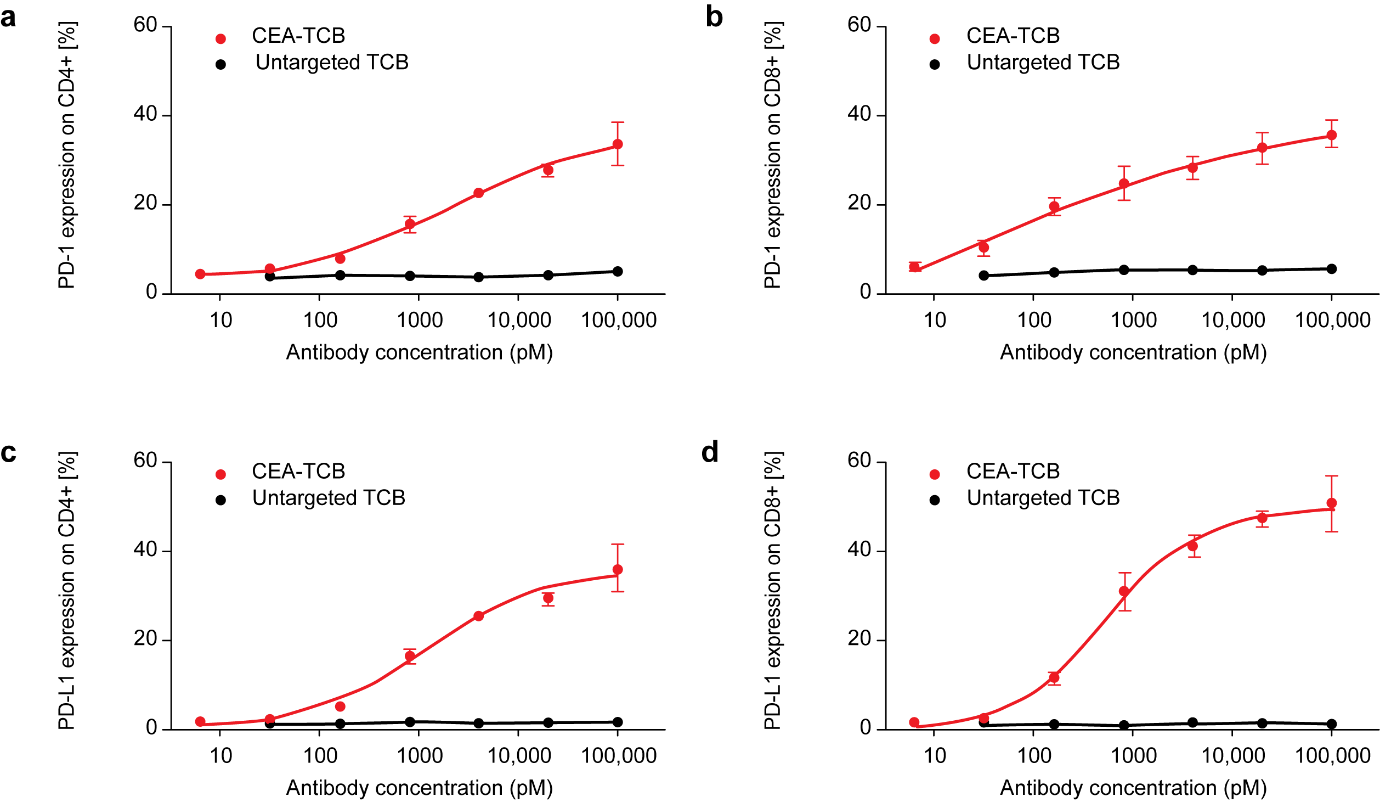


**Supplementary Figure 8.** **CEA-TCB induces the expression of PD-L1 on tumor target cells only in the presence of PBMCs. IFNy blocking inhibits PD-L1 up-regulation on target cells.**

**a**, Upregulation of PD-L1 on tumor cells was higher in the presence of IFNγ. **b+c**, Flow cytometry analysis for PD-L1 expression (MFI) on MKN-45 cells (**b**) and LS174T cells (**c**) after 48 h of incubation with CEA-TCB or untargeted TCB in presence or absence of PBMCs. **d+e** Flow cytometry analysis for PD-L1 expression (MFI) on MKN-45 cells (**d**) and LS174T cells (**e**) recovered after TCB-mediated killing from co-culture assays (48 h) in presence or absence of anti-IFNy antibody. Data are the mean and standard deviation of triplicates.

**
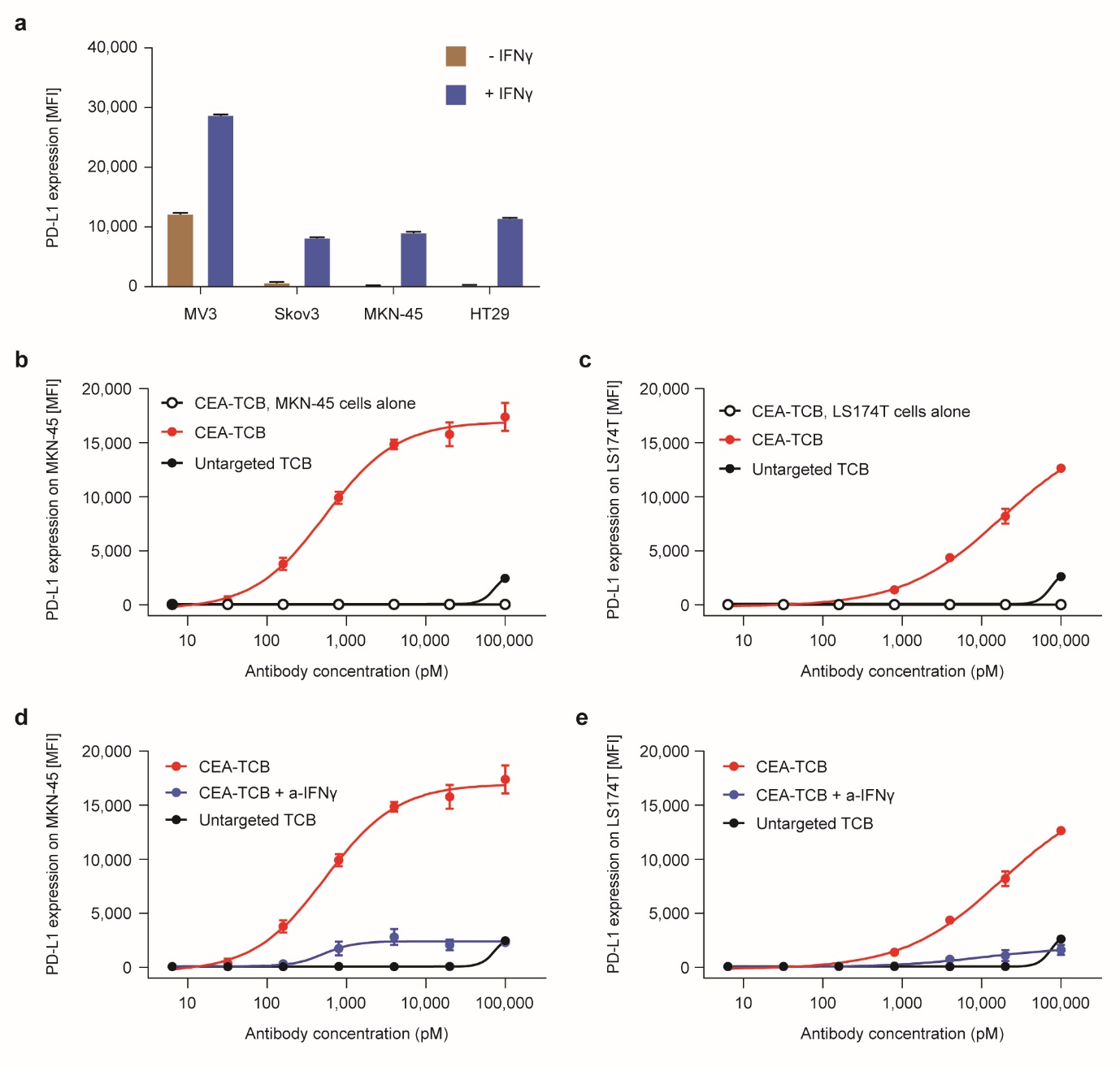
**

**Supplementary Figure 9. Blockade of PD-1/PD-L1 axis improves the efficacy of a suboptimal dose of CD20-TCB in aggressive DLBCL tumor model.**

Hematopoietic stem cell humanized NSG mice were inoculated subcutaneously with 1.5 × 10^6^ WSU-DLCL2 (DLBCL tumor model) cells and treated with either buffer (vehicle), 0.15 mg/kg of CD20-TCB (glofitamab; suboptimal dose for efficacy), 10 mg/kg of a-PD-L1 blocking antibody, or with a combination of CD20-TCB and a-PD-L1 (same dose as in monotherapy), each given i.v. once per week starting with a tumor volume of 520–550 mm^3^ (Day 15). Black arrows indicate treatments (two in total). Tumor growth kinetics are shown as mean ± SEM for all treatment groups (n=9–10 mice per group). Combination group vs CD20-TCB: p=0.021; one-way ANOVA with Tukey’s multiple comparison correction done after two treatments (Day 28).


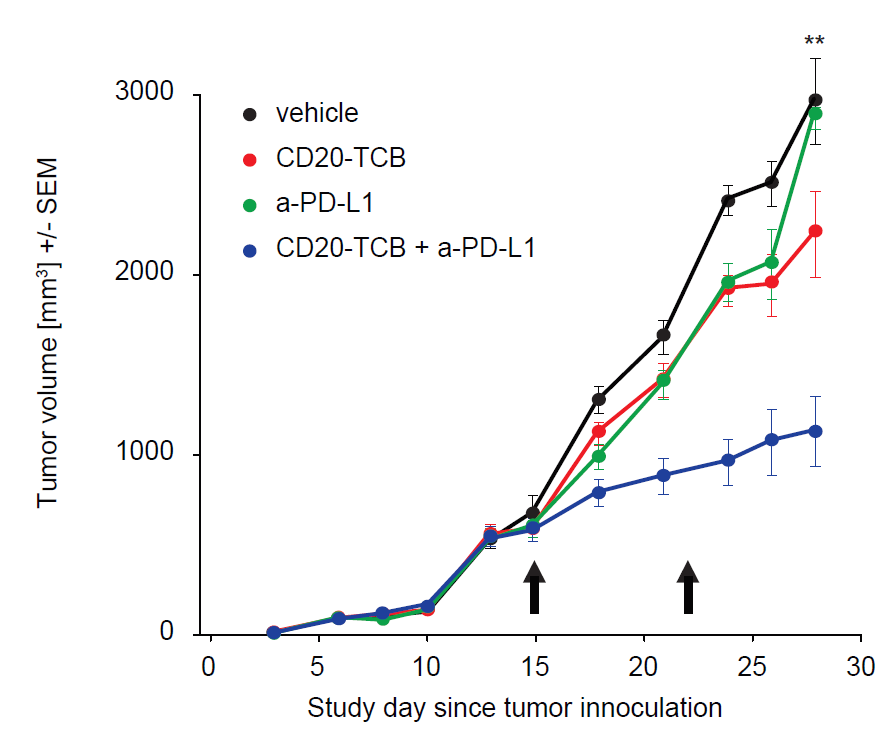


**Supplementary Table 1. Bulk RNA sequencing expression data showing genes that demonstrated significantly different expression between tumors harvested from CEA-TCB-treated and control mice.**

| **HGNC** | **Name** | **log2FC CEA-TCB vs vehicle** | **adj. P-value** |
| --- | --- | --- | --- |
| CXCL13 | chemokine (C-X-C motif) ligand 13 | 6.0403 | 0.0095 |
| GNLY | granulysin | 5.1969 | 0.0010 |
| GZMB | granzyme B (granzyme 2, cytotoxic T-lymphocyte-associated serine esterase 1) | 4.8005 | 0.0081 |
| CXCL10 | chemokine (C-X-C motif) ligand 10 | 4.6972 | 0.0131 |
| IDO1 | indoleamine 2,3-dioxygenase 1 | 4.6867 | 0.0060 |
| SLA | Src-like-adaptor | 4.5353 | 0.0021 |
| CD2 | CD2 molecule | 4.4021 | 0.0016 |
| TRBC2 | T-cell receptor beta constant 2 | 4.3353 | 0.0055 |
| TIGIT | T-cell immunoreceptor with Ig and ITIM domains | 4.2893 | 0.0017 |
| IL2RB | interleukin 2 receptor, beta | 4.1769 | 0.0020 |
| CXCL9 | chemokine (C-X-C motif) ligand 9 | 4.1188 | 0.0110 |
| CX3CL1 | chemokine (C-X3-C motif) ligand 1 | 4.0846 | 0.0051 |
| CCL4L2 | chemokine (C-C motif) ligand 4-like 2 | 4.0320 | 0.0027 |
| CD3E | CD3e molecule, epsilon (CD3-TCR complex) | 4.0205 | 0.0010 |
| UBD | ubiquitin D | 3.6667 | 0.0300 |
| LCP2 | lymphocyte cytosolic protein 2 (SH2 domain containing leukocyte protein of 76kDa) | 3.5571 | 0.0010 |
| CD74 | CD74 molecule, major histocompatibility complex, class II invariant chain | 3.5174 | 0.0128 |
| HLA-DRA | major histocompatibility complex, class II, DR alpha | 3.3482 | 0.0438 |
| GBP1 | guanylate binding protein 1, interferon-inducible | 3.3055 | 0.0118 |
| LCP1 | lymphocyte cytosolic protein 1 (L-plastin) | 3.2974 | 0.0066 |
| IFI16 | interferon, gamma-inducible protein 16 | 3.2475 | 0.0020 |
| CCL5 | chemokine (C-C motif) ligand 5 | 3.1919 | 0.0020 |
| HLA-DRB5 | major histocompatibility complex, class II, DR beta 5 | 3.0907 | 0.0388 |
| PPP1R16B | protein phosphatase 1, regulatory subunit 16B | 2.9882 | 0.0045 |
| NKG7 | natural killer cell group 7 sequence | 2.9718 | 0.0021 |
| PTPRC | protein tyrosine phosphatase, receptor type, C | 2.9174 | 0.0034 |
| CSF1 | colony stimulating factor 1 (macrophage) | 2.8753 | 0.0013 |
| ITGAL | integrin, alpha L (antigen CD11A (p180), lymphocyte function-associated antigen 1; alpha polypeptide) | 2.8681 | 0.0036 |
| SAMD9L | sterile alpha motif domain containing 9-like | 2.8585 | 0.0048 |
| RAC2 | ras-related C3 botulinum toxin substrate 2 (rho family, small GTP binding protein Rac2) | 2.8377 | 0.0057 |
| LAPTM5 | lysosomal protein transmembrane 5 | 2.8318 | 0.0080 |
| ARHGDIB | Rho GDP dissociation inhibitor (GDI) beta | 2.8133 | 0.0099 |
| SRGN | serglycin | 2.7346 | 0.0134 |
| HLA-DRB1 | major histocompatibility complex, class II, DR beta 1 | 2.7262 | 0.0480 |
| NNMT | nicotinamide N-methyltransferase | 2.6549 | 0.0024 |
| CFB | complement factor B | 2.6424 | 0.0057 |
| CD52 | CD52 molecule | 2.6277 | 0.0075 |
| CELF2 | CUGBP, Elav-like family member 2 | 2.5638 | 0.0033 |
| COL1A2 | collagen, type I, alpha 2 | 2.5556 | 0.0048 |
| WIPF1 | WAS/WASL interacting protein family, member 1 | 2.5404 | 0.0063 |
| APOL1 | apolipoprotein L, 1 | 2.4503 | 0.0036 |
| LSP1 | lymphocyte-specific protein 1 | 2.3972 | 0.0091 |
| OTOGL | otogelin-like | 2.3916 | 0.0021 |
| COL9A2 | collagen, type IX, alpha 2 | 2.3789 | 0.0046 |
| ZAP70 | zeta-chain (TCR) associated protein kinase 70kDa | 2.3768 | 0.0033 |
| CD96 | CD96 molecule | 2.3684 | 0.0015 |
| IFI27 | interferon, alpha-inducible protein 27 | 2.2937 | 0.0351 |
| ICAM1 | intercellular adhesion molecule 1 | 2.2467 | 0.0040 |
| GBP5 | guanylate binding protein 5 | 2.2352 | 0.0043 |
| HCLS1 | hematopoietic cell-specific Lyn substrate 1 | 2.2109 | 0.0158 |
| SLC43A3 | solute carrier family 43, member 3 | 2.1830 | 0.0027 |
| ARHGEF6 | Rac/Cdc42 guanine nucleotide exchange factor (GEF) 6 | 2.0466 | 0.0085 |
| IL16 | interleukin 16 | 2.0204 | 0.0010 |
| ITGA1 | integrin, alpha 1 | 2.0126 | 0.0121 |
| PARP15 | poly (ADP-ribose) polymerase family, member 15 | 1.9986 | 0.0058 |
| C3 | complement component 3 | 1.9976 | 0.0027 |
| CYTIP | cytohesin 1 interacting protein | 1.9923 | 0.0021 |
| GBP4 | guanylate binding protein 4 | 1.9862 | 0.0021 |
| PRF1 | perforin 1 (pore forming protein) | 1.9650 | 0.0183 |
| SPOCK2 | sparc/osteonectin, cwcv and kazal-like domains proteoglycan (testican) 2 | 1.9625 | 0.0121 |
| PLEKHS1 | pleckstrin homology domain containing, family S member 1 | 1.9238 | 0.0118 |
| PSMB9 | proteasome (prosome, macropain) subunit, beta type, 9 | 1.8997 | 0.0193 |
| OAS2 | 2'-5'-oligoadenylate synthetase 2, 69/71kDa | 1.8915 | 0.0037 |
| TNFAIP2 | tumor necrosis factor, alpha-induced protein 2 | 1.8825 | 0.0034 |
| IFIT3 | interferon-induced protein with tetratricopeptide repeats 3 | 1.8729 | 0.0137 |
| TRIM22 | tripartite motif containing 22 | 1.8532 | 0.0080 |
| MMP1 | matrix metallopeptidase 1 (interstitial collagenase) | 1.8509 | 0.0056 |
| CXCR6 | chemokine (C-X-C motif) receptor 6 | 1.8139 | 0.0010 |
| HAPLN3 | hyaluronan and proteoglycan link protein 3 | 1.8070 | 0.0021 |
| SLCO1B3 | solute carrier organic anion transporter family, member 1B3 | 1.7627 | 0.0072 |
| SPINK1 | serine peptidase inhibitor, Kazal type 1 | 1.7515 | 0.0215 |
| CYP4X1 | cytochrome P450, family 4, subfamily X, polypeptide 1 | 1.7288 | 0.0081 |
| COL5A2 | collagen, type V, alpha 2 | 1.7180 | 0.0096 |
| DPCR1 | diffuse panbronchiolitis critical region 1 | 1.7030 | 0.0341 |
| PDZK1IP1 | PDZK1 interacting protein 1 | 1.6989 | 0.0143 |
| PIGR | polymeric immunoglobulin receptor | 1.6900 | 0.0314 |
| SFTPA2 | surfactant protein A2 | 1.6700 | 0.0361 |
| PLA2G1B | phospholipase A2, group IB (pancreas) | 1.6640 | 0.0084 |
| RFTN1 | raftlin, lipid raft linker 1 | 1.6543 | 0.0049 |
| LIPC | lipase, hepatic | 1.6491 | 0.0020 |
| PROX1 | prospero homeobox 1 | 1.6429 | 0.0020 |
| C2CD4B | C2 calcium-dependent domain containing 4B | 1.6123 | 0.0040 |
| APOBEC3G | apolipoprotein B mRNA editing enzyme, catalytic polypeptide-like 3G | 1.6060 | 0.0146 |
| CFI | complement factor I | 1.5897 | 0.0078 |
| SLAMF6 | SLAM family member 6 | 1.5629 | 0.0021 |
| CP | ceruloplasmin (ferroxidase) | 1.5572 | 0.0188 |
| RCSD1 | RCSD domain containing 1 | 1.5560 | 0.0080 |
| SFTA2 | surfactant associated 2 | 1.5406 | 0.0037 |
| PTPN7 | protein tyrosine phosphatase, non-receptor type 7 | 1.5337 | 0.0081 |
| ITGB2 | integrin, beta 2 (complement component 3 receptor 3 and 4 subunit) | 1.5311 | 0.0174 |
| SAMD9 | sterile alpha motif domain containing 9 | 1.5271 | 0.0131 |
| KIAA1211L | KIAA1211-like | 1.5226 | 0.0074 |
| MT1E | metallothionein 1E | 1.4754 | 0.0102 |
| ANXA6 | annexin A6 | 1.4661 | 0.0105 |
| IFITM2 | interferon induced transmembrane protein 2 | 1.4598 | 0.0225 |
| APCDD1 | adenomatosis polyposis coli down-regulated 1 | 1.4546 | 0.0260 |
| COL12A1 | collagen, type XII, alpha 1 | 1.4437 | 0.0053 |
| ACAP1 | ArfGAP with coiled-coil, ankyrin repeat and PH domains 1 | 1.4427 | 0.0104 |
| RASSF5 | Ras association (RalGDS/AF-6) domain family member 5 | 1.4292 | 0.0095 |
| KLK6 | kallikrein-related peptidase 6 | 1.4229 | 0.0365 |
| ADAM28 | ADAM metallopeptidase domain 28 | 1.4201 | 0.0041 |
| C8orf4 | chromosome 8 open reading frame 4 | 1.4127 | 0.0429 |
| UBE2L6 | ubiquitin-conjugating enzyme E2L 6 | 1.3909 | 0.0276 |
| ARHGAP30 | Rho GTPase activating protein 30 | 1.3870 | 0.0420 |
| IL10RA | interleukin 10 receptor, alpha | 1.3774 | 0.0072 |
| HAVCR2 | hepatitis A virus cellular receptor 2 | 1.3709 | 0.0077 |
| ETV7 | ets variant 7 | 1.3561 | 0.0293 |
| WIPF3 | WAS/WASL interacting protein family, member 3 | 1.3527 | 0.0220 |
| CCBE1 | collagen and calcium binding EGF domains 1 | 1.3353 | 0.0063 |
| ITGAM | integrin, alpha M (complement component 3 receptor 3 subunit) | 1.3201 | 0.0290 |
| MYO1F | myosin IF | 1.3187 | 0.0410 |
| IL32 | interleukin 32 | 1.3030 | 0.0055 |
| RBPMS2 | RNA binding protein with multiple splicing 2 | 1.3019 | 0.0034 |
| IRF1 | interferon regulatory factor 1 | 1.2998 | 0.0239 |
| SUCNR1 | succinate receptor 1 | 1.2954 | 0.0176 |
| XAF1 | XIAP associated factor 1 | 1.2939 | 0.0058 |
| NEBL | nebulette | 1.2570 | 0.0051 |
| PARP10 | poly (ADP-ribose) polymerase family, member 10 | 1.2562 | 0.0208 |
| CIITA | class II, major histocompatibility complex, transactivator | 1.2396 | 0.0080 |
| C1S | complement component 1, s subcomponent | 1.2233 | 0.0054 |
| PPEF1 | protein phosphatase, EF-hand calcium binding domain 1 | 1.2208 | 0.0036 |
| TNFSF11 | tumor necrosis factor (ligand) superfamily, member 11 | 1.2171 | 0.0175 |
| KRT17 | keratin 17 | 1.2141 | 0.0264 |
| HYAL1 | hyaluronoglucosaminidase 1 | 1.2123 | 0.0151 |
| NPC1L1 | NPC1-like 1 | 1.2026 | 0.0121 |
| FAM84A | family with sequence similarity 84, member A | 1.1993 | 0.0222 |
| LTB | lymphotoxin beta (TNF superfamily, member 3) | 1.1986 | 0.0301 |
| FGFBP1 | fibroblast growth factor binding protein 1 | 1.1941 | 0.0037 |
| TMPRSS3 | transmembrane protease, serine 3 | 1.1854 | 0.0011 |
| LDLRAD4 | low density lipoprotein receptor class A domain containing 4 | 1.1642 | 0.0369 |
| GNAI1 | guanine nucleotide binding protein (G protein), alpha inhibiting activity polypeptide 1 | 1.1555 | 0.0249 |
| XG | Xg blood group | 1.1538 | 0.0332 |
| CORO1A | coronin, actin binding protein, 1A | 1.1396 | 0.0183 |
| SERPINA1 | serpin peptidase inhibitor, clade A (alpha-1 antiproteinase, antitrypsin), member 1 | 1.1375 | 0.0154 |
| GAD1 | glutamate decarboxylase 1 (brain, 67kDa) | 1.1307 | 0.0080 |
| LAG3 | lymphocyte-activation gene 3 | 1.1295 | 0.0152 |
| CHI3L2 | chitinase 3-like 2 | 1.1231 | 0.0383 |
| CXCL2 | chemokine (C-X-C motif) ligand 2 | 1.1212 | 0.0120 |
| TYMP | thymidine phosphorylase | 1.1094 | 0.0164 |
| PHACTR3 | phosphatase and actin regulator 3 | 1.1005 | 0.0024 |
| PPP1R1A | protein phosphatase 1, regulatory (inhibitor) subunit 1A | 1.0947 | 0.0056 |
| RBM20 | RNA binding motif protein 20 | 1.0900 | 0.0198 |
| LIMD2 | LIM domain containing 2 | 1.0860 | 0.0178 |
| IFFO1 | intermediate filament family orphan 1 | 1.0702 | 0.0071 |
| IFI6 | interferon, alpha-inducible protein 6 | 1.0549 | 0.0381 |
| KYNU | kynureninase | 1.0527 | 0.0225 |
| CCND2 | cyclin D2 | 1.0518 | 0.0431 |
| PPP1R3C | protein phosphatase 1, regulatory subunit 3C | 1.0479 | 0.0242 |
| MTURN | maturin, neural progenitor differentiation regulator homolog (Xenopus) | 1.0466 | 0.0054 |
| BATF2 | basic leucine zipper transcription factor, ATF-like 2 | 1.0436 | 0.0365 |
| MFSD7 | major facilitator superfamily domain containing 7 | 1.0396 | 0.0480 |
| BEX5 | brain expressed, X-linked 5 | 1.0363 | 0.0192 |
| TNFRSF18 | tumor necrosis factor receptor superfamily, member 18 | 1.0355 | 0.0113 |
| IFIT2 | interferon-induced protein with tetratricopeptide repeats 2 | 1.0338 | 0.0348 |
| CXCR4 | chemokine (C-X-C motif) receptor 4 | 1.0336 | 0.0172 |
| FGF18 | fibroblast growth factor 18 | 1.0211 | 0.0041 |
| THBS1 | thrombospondin 1 | 1.0165 | 0.0214 |
| GRIN2D | glutamate receptor, ionotropic, N-methyl D-aspartate 2D | 1.0155 | 0.0078 |
| MX1 | myxovirus (influenza virus) resistance 1, interferon-inducible protein p78 (mouse) | 1.0100 | 0.0468 |
| PTGS1 | prostaglandin-endoperoxide synthase 1 (prostaglandin G/H synthase and cyclooxygenase) | 1.0096 | 0.0063 |
| RAB31 | RAB31, member RAS oncogene family | 1.0008 | 0.0055 |
| CA13 | carbonic anhydrase XIII | -1.0023 | 0.0026 |
| LPHN3 | latrophilin 3 | -1.0049 | 0.0179 |
| QPCT | glutaminyl-peptide cyclotransferase | -1.0520 | 0.0214 |
| SYT13 | synaptotagmin XIII | -1.0594 | 0.0122 |
| MYBPC1 | myosin binding protein C, slow type | -1.0603 | 0.0031 |
| PSD2 | pleckstrin and Sec7 domain containing 2 | -1.0665 | 0.0113 |
| IGFBP6 | insulin-like growth factor binding protein 6 | -1.0697 | 0.0257 |
| NXF3 | nuclear RNA export factor 3 | -1.0787 | 0.0334 |
| KCNH4 | potassium voltage-gated channel, subfamily H (eag-related), member 4 | -1.0799 | 0.0126 |
| TGFBI | transforming growth factor, beta-induced, 68kDa | -1.0847 | 0.0111 |
| SPRR3 | small proline-rich protein 3 | -1.0881 | 0.0356 |
| THRB | thyroid hormone receptor, beta | -1.0922 | 0.0034 |
| EPHA8 | EPH receptor A8 | -1.0923 | 0.0360 |
| EEF1A2 | eukaryotic translation elongation factor 1 alpha 2 | -1.1001 | 0.0102 |
| F7 | coagulation factor VII (serum prothrombin conversion accelerator) | -1.1180 | 0.0063 |
| CLCN1 | chloride channel, voltage-sensitive 1 | -1.1265 | 0.0335 |
| PKD2L2 | polycystic kidney disease 2-like 2 | -1.1416 | 0.0391 |
| PABPC3 | poly(A) binding protein, cytoplasmic 3 | -1.1510 | 0.0261 |
| FIBIN | fin bud initiation factor homolog (zebrafish) | -1.1902 | 0.0335 |
| FGD5 | FYVE, RhoGEF and PH domain containing 5 | -1.2041 | 0.0021 |
| RAB3IL1 | RAB3A interacting protein (rabin3)-like 1 | -1.2249 | 0.0260 |
| CACNA1D | calcium channel, voltage-dependent, L type, alpha 1D subunit | -1.2297 | 0.0045 |
| EDN3 | endothelin 3 | -1.2373 | 0.0112 |
| ZNF880 | zinc finger protein 880 | -1.2419 | 0.0218 |
| UNC93A | unc-93 homolog A (C. elegans) | -1.2430 | 0.0413 |
| PTPRR | protein tyrosine phosphatase, receptor type, R | -1.2580 | 0.0085 |
| COL26A1 | collagen, type XXVI, alpha 1 | -1.2605 | 0.0281 |
| NFASC | neurofascin | -1.2637 | 0.0457 |
| KLK1 | kallikrein 1 | -1.3086 | 0.0050 |
| BAIAP3 | BAI1-associated protein 3 | -1.3102 | 0.0382 |
| SPON2 | spondin 2, extracellular matrix protein | -1.3113 | 0.0463 |
| INHBA | inhibin, beta A | -1.3154 | 0.0209 |
| TMEM150B | transmembrane protein 150B | -1.3594 | 0.0084 |
| UGT2B15 | UDP glucuronosyltransferase 2 family, polypeptide B15 | -1.3630 | 0.0420 |
| EDA2R | ectodysplasin A2 receptor | -1.3687 | 0.0086 |
| ASIC4 | acid-sensing (proton-gated) ion channel family member 4 | -1.3815 | 0.0348 |
| LRAT | lecithin retinol acyltransferase (phosphatidylcholine--retinol O-acyltransferase) | -1.3963 | 0.0067 |
| ZNF429 | zinc finger protein 429 | -1.4256 | 0.0327 |
| C21orf88 | chromosome 21 open reading frame 88 | -1.5015 | 0.0104 |
| DSC3 | desmocollin 3 | -1.5024 | 0.0153 |
| NTN4 | netrin 4 | -1.5168 | 0.0354 |
| AZGP1 | alpha-2-glycoprotein 1, zinc-binding | -1.5255 | 0.0447 |
| C20orf195 | chromosome 20 open reading frame 195 | -1.6171 | 0.0218 |
| POU4F1 | POU class 4 homeobox 1 | -1.6768 | 0.0183 |
| DHRS9 | dehydrogenase/reductase (SDR family) member 9 | -1.7387 | 0.0209 |
| SCN4B | sodium channel, voltage-gated, type IV, beta subunit | -1.7541 | 0.0104 |
| AKR1B10 | aldo-keto reductase family 1, member B10 (aldose reductase) | -1.8578 | 0.0288 |
| KRT20 | keratin 20 | -1.9430 | 0.0141 |
| KRT6A | keratin 6A | -1.9617 | 0.0297 |
| CORO2B | coronin, actin binding protein, 2B | -2.2493 | 0.0062 |
